# Parasite-Derived Excretory-Secretory Products Alleviate Gut Microbiota Dysbiosis and Improve Cognitive Impairment Induced by a High-Fat Diet

**DOI:** 10.3389/fimmu.2021.710513

**Published:** 2021-10-20

**Authors:** Jiacheng Wu, Yuqi Zhu, Limian Zhou, Yang Lu, Tingting Feng, Mengyu Dai, Jiaxue Liu, Wen Xu, Wanpeng Cheng, Fenfen Sun, Hua Liu, Wei Pan, Xiaoying Yang

**Affiliations:** ^1^ Jiangsu Key Laboratory of Immunity and Metabolism, Department of Pathogen Biology and Immunology, Xuzhou Medical University, Xuzhou, China; ^2^ The Second School of Clinical Medicine, Xuzhou Medical University, Xuzhou, China; ^3^ National Demonstration Center for Experimental Basic Medical Science Education, Xuzhou Medical University, Xuzhou, China; ^4^ The First School of Clinical Medicine, Xuzhou Medical University, Xuzhou, China; ^5^ The School of Anesthesiology, Xuzhou Medical University, Xuzhou, China; ^6^ National Institute of Parasitic Diseases, Chinese Center for Disease Control and Prevention (Chinese Center for Tropical Diseases Research); National Health Commission (NHC) Key Laboratory of Parasite and Vector Biology; WHO Collaborating Centre for Tropical Diseases; National Center for International Research on Tropical Diseases, Shanghai, China

**Keywords:** *Echinococcus granulosus*, excretory-secretory products, microbiota-gut-brain axis, cognition, obesity, gut microbiota, neuroinflammation

## Abstract

High-fat (HF) diet-induced neuroinflammation and cognitive decline in humans and animals have been associated with microbiota dysbiosis *via* the gut-brain axis. Our previous studies revealed that excretory-secretory products (ESPs) derived from the larval *Echinococcus granulosus* (*E. granulosus*) function as immunomodulators to reduce the inflammatory response, while the parasitic infection alleviates metabolic disorders in the host. However, whether ESPs can improve cognitive impairment under obese conditions remain unknown. This study aimed to investigate the effects of *E. granulosus*-derived ESPs on cognitive function and the microbiota-gut-brain axis in obese mice. We demonstrated that ESPs supplementation prevented HF diet-induced cognitive impairment, which was assessed behaviorally by nest building, object location, novel object recognition, temporal order memory, and Y-maze memory tests. In the hippocampus (HIP) and prefrontal cortex (PFC), ESPs suppressed neuroinflammation and HF diet-induced activation of the microglia and astrocytes. Moreover, ESPs supplementation improved the synaptic ultrastructural impairments and increased both pre- and postsynaptic protein levels in the HIP and PFC compared to the HF diet-treated group. In the colon, ESPs reversed the HF diet-induced gut barrier dysfunction, increased the thickness of colonic mucus, upregulated the expression of zonula occludens-1 (ZO-1), attenuated the translocation of bacterial endotoxins, and decreased the colon inflammation. Notably, ESPs supplementation alleviated the HF diet-induced microbiota dysbiosis. After clarifying the role of antibiotics in obese mice, we found that broad-spectrum antibiotic intervention abrogated the effects of ESPs on improving the gut microbiota dysbiosis and cognitive decline. Overall, the present study revealed for the first time that the parasite-derived ESPs alleviate gut microbiota dysbiosis and improve cognitive impairment induced by a high-fat diet. This finding suggests that parasite-derived molecules may be used to explore novel drug candidates against obesity-associated neurodegenerative diseases.

## Introduction

Obesity, a global epidemic and systemic metabolic disease that endangers human health, is a serious public health concern (1). In fact, there is accumulating evidence that obesity is an important risk factor for neurodegenerative diseases, such as Alzheimer’s disease (AD) (2, 3). Poor diet and obesity act through multiple mechanisms to affect cognitive health including neuroinflammation, alternation of gut microbiome composition, neurotrophic factors, neurogenesis, ultrastructural damage to synapses and oxidative stress (4). As a result, challenge for future research is to identify the key processes that can be reversed to improve cognition. Neuroinflammation, a common hallmark of neurodegenerative diseases, is closely linked to cognitive decline and obesity (2, 5, 6). Microglia and astrocytes, resident immune cells of the central nervous system, are extensively activated under neuroinflammatory conditions, resulting in synaptic damage and cognitive impairment (2, 5–8). There is growing evidence regarding the crucial role of neuroinflammation in the development of cognitive dysfunction in obesity (5, 9, 10). For example, chronic HF diet reduces the tight junction protein levels, while inducing gliosis (activation of microglia and astrocytes) and inflammation in the brain, thereby causing neurodegeneration. Our previous studies demonstrated that dietary β-glucan could constrain microglial activation, reduce neuroinflammation, and consequently improve cognitive impairment in obese mice (10). Therefore, alleviating neuroinflammation mediated by immune cells in the brain may be a promising strategy for the treatment of neurodegenerative diseases.

The gut microbiota plays significant roles in brain function and behavior *via* the gut-brain axis. Further, dysbiosis of the microbiota is closely related to neuroinflammation and cognitive decline (11–13). It has been reported that the decrease in the richness and diversity of gut microbiota in obese mice induced by HF diet is accompanied by a decrease in the expression levels of the intestinal tight junction proteins, thereby resulting in gut barrier dysfunction. Consequently, increased intestinal permeability allows hyper-translocation of lipopolysaccharides into the blood, causing endotoxemia and eventually inducing cognitive decline (14). Furthermore, transplantation of obese-type gut microbiota has been reported to impair intestinal function, induce neuroinflammation, and even cause neurobehavioral changes in mice (15). Previous studies in germ-free and antibiotic-treated specific pathogen-free rodents revealed that gut microbiota dysbiosis activates microglia and consequently influences hippocampal neurogenesis and brain development (16). Therefore, dysregulation of the gut-brain axis is considered a crucial factor for the cognitive impairment induced by a chronic HF diet. Consequently, dietary probiotics or prebiotics could improve cognitive impairment *via* the gut-brain axis (12). Aligning with these findings, sodium oligomannate, a phase III clinical trial drug for AD, has recently been reported to show solid and consistent cognitive improvement *via* remodeling of the gut microbiota (17). Given the close relationship between gut microbiota imbalance, neuroinflammation, and cognitive dysfunction, gut microbiome remodeling has been proposed as a promising therapeutic strategy for cognitive impairment (18, 19).

Parasites, which are pathogens that feed on nutrients obtained from the hosts, exhibit high infection rates and low mortality rates in humans and animals. Accumulating evidence shows that parasites have developed refined strategies to manipulate the anti-infective immunity of the host, thereby enabling them to coexist with their hosts for a long time (20, 21). Population epidemiological data from recent years show that parasitic infections are negatively correlated with the incidence of obesity and metabolic diseases (22, 23). However, once the parasites are completely expelled by drugs, the population will have a higher incidence ratio of immunometabolic diseases (24). As a result, researchers began to utilize several live parasites to explore new intervention strategies for human diseases (25, 26). As live parasite therapy may pose certain biosafety issues (27), the excretory-secretory products (ESPs), important mixtures exploited by parasites to modify host immunity (28–30), have become ideal targets for developing parasite-based therapies against obesity (29); however, whether parasite-derived ESPs can improve obesity-induced cognitive impairment has not been reported.

*Echinococcus granulosus* (*E. granulosus*) is a cestode known for its high host adaptability and worldwide distribution. Accumulating evidence shows that larval *E. granulosus* has evolved novel strategies to subvert immune responses of the host (31). Parasitic infection can induce the accumulation of immunosuppressive cells to downregulate the anti-infective immunity of the host (32–35). Moreover, ESPs derived from parasites can directly inhibit the differentiation of T helper 17 (Th17) cells and polarize macrophages toward the M2 phenotype (34, 36), thereby creating an anti-inflammatory microenvironment. In addition, our latest study showed that the larval *E. granulosus* infection enhances lipolysis, which is accompanied by a decline in carbohydrate oxidation and an increase in fatty oxidation (37). These studies collectively suggest that larval *E. granulosus* and its ESPs can effectively remodel the host immunity and metabolism. Given that both inflammation and metabolic disorders contribute to obesity and related cognitive impairment, we speculated that the ESPs of larval *E. granulosus* may have the potential to alleviate cognitive impairment.

In the current study, a HF diet mouse model was employed to induce cognitive impairment and subsequently evaluate the effects of *E. granulosus*-derived ESPs on various parameters, including the composition of gut microbiota, thickness of colonic mucus, levels of tight junction proteins and serum lipopolysaccharides (LPS). Moreover, the pro-cognitive efficacy of ESPs in this model was evaluated by examining the synaptic ultrastructure and neuroinflammation of the hippocampus (HIP) and prefrontal cortex (PFC), which are two key areas involved in cognition. In addition, an antibiotic intervention test was used to assess the possibility of a causal relationship between the ESPs-induced changes in gut microbiota and effects on cognition. The present study demonstrates for the first time that parasite-derived ESPs can ameliorate gut microbiota dysbiosis and protect against cognitive impairment in diet-induced obesity *via* the gut-brain axis. This finding provides a novel strategy for developing parasite-based therapies against obesity-related neurodegenerative diseases.

## Materials and Methods

### ESPs Collection of Larval *E. granulosus*

The ESPs derived from the larval *E. granulosus* were prepared as previously described (34, 35, 38). Hydatid cysts were collected from the livers of a naturally infected sheep in a slaughterhouse in Qinghai, China. Cyst fluids containing protoscoleces (PSCs) were sucked out of the cysts using a sterile syringe. *E. granulosus* PSCs obtained from the hydatid cysts were subject to a cycle of washings through successive sedimentations in phosphate buffer saline (PBS), so that to eliminate the remaining hydatid debris. The genotype of the *E. granulosus* protoscoleces used in this study was sheep G1. Then, 10,000 *E. granulosus* PSCs were cultured in 2 ml of sterile normal saline supplemented with 10% glucose, 100 μg/ml penicillin and 100 U/ml streptomycin (Invitrogen, USA) in 6-well plates at 37°C in 5% CO_2_. The supernatants containing ESPs released by the *E. granulosus*, but without the parasites, were renewed every 8 h and concentrated using Ultrafree 15 filters with a 5 kDa pore diameter membrane (Millipore, Watford, USA). EDTA (5 mM/l) and phenyl methyl sulfonyl fluoride (PMSF, 2 mM/l), as enzymatic inhibitors, were added and collected as native ESPs of the larval *E. granulosus*. The concentration of ESPs was measured using the bicinchoninic acid (BCA) protein concentration assay kit (Beyotime Biotech, Beijing, China). The endotoxin in ESPs was carefully removed according to the protocol of Solution Endotoxin Erasol Kit (TIAN, Beijing, China). The collected ESPs were stored in -80°C. Before use, ESPs were diluted in a vehicle control at a concentration of 100 μg/ml. Moreover, 10% glucose solution treated with EDTA and PMSF acts as the vehicle control. At each time of renewing supernatants, the PSCs viability was determined by staining with 0.1% eosin and observed under light microscope. Only PSCs with > 95% viability were used for preparing the ESPs.

### Animals and Treatment

Male C57BL/6J mice (8 weeks old) were obtained from Shanghai Laboratory Animal Center (SLAC) and were bred in the Experimental Animal Center of Xuzhou Medical University. All mice were housed in an air-conditioned room at 24°C with a 12 h dark/light cycle and permitted free access to standard laboratory food and water. After habituation to the laboratory environmental for 1 week, the mice were matched according to fat mass and body weight and randomly divided into four groups (n=12 per group): (1) mice fed a low-fat (LF) diet (5% fat by weight) and intraperitoneally injected with 200 μl vehicle control twice a week as the LF group; (2) mice fed a LF diet (5% fat by weight) and intraperitoneally injected with 200 μl vehicle control which was added with 20 μg ESPs twice a week as the LFE group; (3) mice receiving the HF diet (60% fat by weight) and intraperitoneally injected with 200 μl vehicle control twice a week as the HF group; (4) mice receiving the HF diet (60% fat by weight) and intraperitoneally injected with 200 μl vehicle control which was added with 20 μg ESPs twice a week as the HFE group. In addition, another two groups (n=12 per group) paralleled with HF group and HFE group were given a cocktail of antibiotics (HF+Ab, HFE+Ab) in the drinking water to investigate the role of gut microbiota in ESPs intervention. Two days prior to HF diet initiation, the HF+Ab and HFE+Ab mice were given drinking water containing ampicillin (1 g/L), vancomycin (0.25 g/L), neomycin (1 g/L), and metronidazole (1 g/L), which was prepared fresh every 3 days. All the groups were given intervention for 12 weeks followed by cognitive behavior tests. The cognitive behavior tests were performed (N = 12 per group), including the nesting behavioral test, object location test at 13^th^ week of feeding, the novel object recognition test and Y-maze memory test at 14^th^ week of feeding and the temporal order memory test at 15^th^ week of feeding. Mice were then anesthetized with chloral hydrate and sacrificed after measuring the body weight and behavioral testing. Liver and fat pads (subcutaneous, epididymal, and brown) were dissected and weighed. Blood serum, intestinal and brain tissues were collected and stored in -80°C for further analyses.

### Behavioral Testing

The nesting behavior test was performed based on methods previously described (10, 39), to evaluate spontaneous rodent behavior. In brief, during the nesting behavior test, the deacon nest score and untore nestlet weight were used to evaluate the activities of daily living typically altered in patients with cognitive impairment. The deacon nest score was scored according to a previously described scoring system, on a definitive 5-point nest-rating scale (39).

The object location test was performed based on methods previously described (10, 40). Briefly, there are three stages in the object location test. The first stage is habituation, in which a mouse was allowed to explore the open field for 5 min. After 24 h, beginning the training stage, in which the mouse allowed to explore the arena for 5 min with 2 identical objects placed parallel. After 1 h, retention session takes place. Mice were allowed to explore the arena with one of the objects remaining in the same location as in trial 2 and the second object moved to a new location. The place discrimination index (PDI) was calculated by using the formula: the time spent with the object moved to a novel place/the total time spent in exploring both the object moved to a novel place and the object remaining in the familiar place × 100.

The novel object recognition test (ORT) was performed based on methods previously described (10, 41, 42). Briefly, there are three stages in the ORT. The first stage is habituation, in which a mouse was allowed to explore the open field for 5 min. After 24 h, beginning the training stage, in which the mouse allowed to explore the arena for 5 min with 2 identical objects placed parallel. After 1 h, retention session takes place. Mice were allowed to explore the arena with one of the familiar objects and one novel object placed parallel for 5 min. The discrimination index was evaluated by using the formula [Time with recent object/(Time with the older object + Time with recent object)] × 100.

The temporal order memory test was performed based on methods previously described (41, 42). In brief, the experiment comprised two sample trials and one test trial with an inter-trial interval of 60 min between each trial. Place mice in behavioral testing room 1 h before the test so they can acclimatize to the conditions. In each sample trial, the mice were allowed to explore two copies of the same object for 4 min; the objects were different between the two sample trials (sample trial 1: object A and A’; sample trial 2: object B and B’). During the test trial, one object from sample trial 1 (A; old familiar) and another object from sample trial 2 (B; recent familiar) were presented parallel and mice were allowed to explore the open field undisturbed for 3 min. A discrimination ratio was calculated by using the formula [(old familiar time − recent familiar time)/total exploration time]. Intact object recognition memory for temporal order was considered if the mice spent more time exploring the old familiar object compared with the recent familiar object.

The Y-maze memory test was performed based on methods previously described (43). After acclimatization of the mice, label the arms of the maze with different pictures. The experiment comprised two sample trials, one arm was closed (novel arm) before mice were placed individually into one of the other two arms (start arm) facing the arm end in a quasi-randomized order during the training trial. Animals were allowed to explore both arms (start and familiar arm) for 5 min. After an inter-trial interval of 1 h, test animals were returned to the Y-maze and allowed to explore all three arms of the maze (start, familiar, and novel) for 5 min. the ratio of time spent in novel arm (%) was calculated by using the formula: (the time spent in novel arm/the total exploration time) × 100.

The sample size was determined by power analysis on website (http://powerandsamplesize.com/Calculators/Compare-k-Means/1-Way-ANOVA-Pairwise) at the liberal significance level of α=0.05 (two-sided) and the power of 1-β=80%. The sample size n=12 each group was estimated based on the data of five behavior tests in the previous intervention (10, 41, 44): mean1 = 0.2, mean2 = 0.75, SD=0.3, power of 1-β=0.9879 in temporal order memory test; mean1 = 0.4, mean2 = 0.6, SD=0.1, power of 1-β=0.9961 in novel objective test; mean1 = 1.8, mean2 = 3.6, SD=0.8, power of 1-β=0.9995 in nesting behavior test; mean1 = 38, mean2 = 52, SD=8, power of 1-β=0.9798 in object location test; mean1 = 39, mean2 = 32, SD=5, power of 1-β=0.8834 in Y-maze memory test.

### Gut Microbiota Analysis

Fecal samples were collected from all mice upon defecation at the end point (week 15) and were stored at -80°C for the subsequent analyses. 16S rRNA gene-based analysis was used to detect the fecal microbiota composition in mice. The hexadecyltrimethylammonium bromide (CTAB) method of Doyle and Doyle (1987), with modifications as described by Griffith and Shaw (1998), was used to extract total genomic DNA from the samples (45). CTAB was purchased from Tianjin Guangfu Fine Chemical Research Institute. The concentration and purity of the DNA were monitored with 1% agarose gels, and the DNA was diluted to 1 ng/L. The V4 region of the 16S rRNA genes were amplified used specific primer (515F806R) with the barcode. All polymerase chain reactions were carried out with 15 µL of Phusion^®^ High-Fidelity PCR Master Mix (New England Biolabs); 0.2 µM of forward and reverse primers, and about 10 ng template DNA. Thermal cycling consisted of initial denaturation at 98°C for 1 min, followed by 30 cycles of denaturation at 98°C for 10 s, annealing at 50°C for 30 s, and elongation at 72°C for 30 s. Finally, 72°C for 5 min. Mix same volume of 1X loading buffer (contained SYB green) with PCR products and operate electrophoresis on 2% agarose gel for detection. PCR products were mixed in equidensity ratios. Then, mixture PCR products were purified with Qiagen Gel Extraction Kit (Qiagen, Germany). Sequencing libraries were generated usingTruSeq^®^ DNA PCR-Free Sample Preparation Kit (Illumina, USA) following manufacturer’s recommendations and index codes were added. The library quality was assessed on the Qubit@ 2.0 Fluorometer (Thermo Scientific) and Agilent Bioanalyzer 2100 system. At last, the library was sequenced on an Illumina NovaSeq platform and 250 bp paired-end reads were generated. Paired-end reads was assigned to samples based on their unique barcode and truncated by cutting off the barcode and primer sequence. Paired-end reads were merged using Fast Length Adjustment of SHort reads (FLASH) (V1.2.7, http://ccb.jhu.edu/software/FLASH/), which was designed to merge paired-end reads when at least some of the reads overlap the read generated from the opposite end of the same DNA fragment, and the splicing sequences were called raw tags. Quality filtering on the raw tags were performed under specific filtering conditions to obtain the high-quality clean tags according to the Quantitative Insights Into Microbial Ecology (QIIME) (V1.9.1, http://qiime.org/scripts/split_libraries_ fastq.html) quality controlled process. The tags were compared with the reference database (Silva database, https://www.arb-silva.de/) using UCHIME algorithm (UCHIME Algorithm, http://www.drive5.com/usearch/manual/uchime_algo.html) to detect chimera sequences, and then the chimera sequences were removed. Then the effective tags finally obtained. Sequences analysis was performed by Uparse software (Uparse v7.0.1001, http://drive5.com/uparse/). Sequences with ≥97% similarity were assigned to the same OTUs. Representative sequence for each OTU was screened for further annotation. For each representative sequence, the Silva Database (http://www.arb-silva.de/) was used based on Mothur algorithm to annotate taxonomic information. The linear discriminant analysis (LDA) effect size (LEfSe) was used to detect the features of gut microbiota with significant difference abundances between designated taxa with the Kruskal−Wallis ranksum test.

### LPS Determination

LPS levels in sera were detected using a chromogenic end-point TAL kit (Xiamen Bioendo Technology Co., Ltd, Xiamen, China) according to the manufacturer’s protocol. The absorbance was determined at a wavelength of 545 nm using a spectrophotometer (Asuragen ClinBio128, USA). All samples for LPS measurements were performed in duplicate.

### Thickness Measurements of the Colonic Mucus Layer

Post Carnoy’s fixation, methanol-stored colon samples were embedded in paraffin, cut into thin sections (5 μm), and mounted on glass slides. Alcian blue staining was performed as previously described (10). The sections were deparaffinized in xylene and hydrated to distilled water. Next, sections were incubated with alcian blue solution for 30 minutes and washed in running tap water for 2 minutes. After being rinsed in distilled water, sections were dehydrated (2× changes) and treated with absolute alcohol (2× changes), 3 minutes each. At last, they were cleared in 3 changes of xylene for 3 minutes each and after which the cover glass was mounted. The thickness of the colonic sections was measured (2 measurements per section/2 sections per animal/3 animals per group) using Image J.

### Immunofluorescence

ZO-1 and F4/80 staining of the colon was detected by staining the colonic tissue sections (5 μm) with anti-ZO-1 antibody (Servicebio, GB11195, 1:200 dilution) in PBS, anti-F4/80 antibody (Servicebio, GB11027, 1:1000 dilution) in PBS and goat-anti-rabbit CY3 conjugated antibody (Servicebio, GB21303, 1:300 dilution) in PBS. Finally, the sections were counterstained with DAPI (Servicebio, G1012). At a temperature of − 18°C, 20 μm frozen brain sections were cut using a cryostat. The brain slices were blocked with 3% bovine serum albumin for 30 min at room temperature and then incubated with the primary antibodies at 4°C overnight. The primary antibody anti-Iba1 (Servicebio, GB13105, 1:500 dilution) and anti-GFAP (Servicebio, GB11096, 1:800 dilution) were used. After washing with PBS, the sections were incubated with the secondary antibodies at 37°C for 50 min. The secondary antibody goat-anti-rabbit Cy3 conjugated antibody (Servicebio, GB21303, 1:300 dilution) were used. Finally, the sections were counterstained with DAPI (Servicebio, G1012) and then imaged with microscope (Nikon Eclipse C1). Quantification of positively stained cells in the PFC and HIP regions were used ImageJ.

### Transmission Electron Microscopy (TEM)

After transcardial perfusion with saline, brain tissues were taken out and 1 mm^3^ of tissue blocks from the CA1 region was dissected. Samples were fixed in a 2% paraformaldehyde-2.5% glutaraldehyde mixture for 24 h and treated post-fixation with 1% osmium tetroxide (OsO4) for 2 h, before dehydration in an ascending graded ethanol series and embedding in epoxy resin. Sections (70 nm) were cut and stained with 4% uranyl acetate and 0.5% lead citrate. Ultrastructure of synapses in the PFC was measured under a transmission electron microscope (Hitachi HT7800 TEM, Japan), and synaptic morphometrics were studied. Gray type I synapses (asymmetric synapses considered to mediate excitatory transmission) were identified in the micrographs by the presence of synaptic vesicles (SVs) and dense material in postsynaptic axon terminal. The software imageJ was used to analyze the microstructural parameters including the length of active zone (AZ), the postsynaptic density (PSD) thickness (measured the thickest part), the width of the synaptic clefts (SC) (estimated by measuring the widest and narrowest portions of the synapse and then averaging these values) and the synaptic curvature (expressed by the ratio of synaptic post interface arch length and chord length).

### RNA Extraction and Quantitative (q) Real-Time PCR (qPCR)

Total RNA was extracted with Trizol (Thermo Fisher Scientific, USA) from the colon, HIP, and PFC. The total RNA concentration was confirmed using a spectrophotometer (DU800, Beckman Coulter Inc., Brea, CA, USA) at 260 nm and 280 nm. RNA purity was determined by the absorption ratios (260/280 nm), which were 1.8–2.0 for all samples. RNA integrity was detected by 1% agarose gel electrophoresis (**Supplementary Figure S1**). Then, 1 μg purified RNA was reverse-transcripted to cDNA using a high-capacity cDNA reverse transcription kit (Takara, Japan). qPCR was performed using the SYBR GREEN Master Mix (TaKaRa, Japan) and determined on a real-time PCR detection system (Bio-Rad, United States). Finally, the CT value of each reaction was provided and the changes in transcriptional level of target gene normalized to β-actin were calculated by the following formula: Relative mRNA level of target gene (folds of control) = 2^−ΔΔCT^. Primer sequences were as follows: mTNFα–forward (F): CTTGTTGCCTCCTCTTTTGCTTA, mTNFα–reverse (R): CTTTATTTCTCTCAATGACCCGTAG; mIL-1β–forward (F): TGGGAA ACAACAGTGGTCAGG, mIL-1β–reverse (R): CTGCTCATTCACGAAAAGGGA; mIL-6–forward (F): TCACAGAAGGAGTGGCTAAGGACC, mIL-6–reverse (R): ACGCACTAGGTTT GCCGAGTAGAT; mβ-actin–forward (F): CGTGGGCCGCCCTAGGCACCA, mβ-actin–reverse (R): TTGGCCTTAGGGTTCAGGGGGG; mCD68–forward (F): TCACCTTGACCTGCTCTCT CTAA, mCD68–reverse (R): GCTGGTAGGTTGATTGTCGTCTG.

### Western Blotting

Mouse colon, HIP and PFC were homogenized in ice-cold RIPA lysis buffer, supplemented with complete EDTA-free protease inhibitor cocktail and PhosSTOP Phosphatase Inhibitor. The homogenate was sonicated six times for 4 s, at 6 s intervals on ice and then centrifuged at 12,000 *g* for 20 min at 4°C. The supernatant was collected, and the protein concentration was quantitated by BCA assay. Equal amounts of protein were separated by sodium dodecyl sulfate-polyacrylamide gel electrophoresis (SDS-PAGE) and transferred onto polyvinylidene difluoride (PVDF) membranes. The membrane was blocked with 5% non-fat milk at room temperature for 1 h, and then incubated with the primary antibody at 4°C overnight. These primary antibodies were included: brain derived neurotrophic factor (BDNF) antibody (Alomone labs, ANT-010), postsynaptic density-95 (PSD-95) antibody (Cell Signaling Technology, #3450), synaptophysin (synaptophysin) antibody (Abcam, ab32127), TNF-α antibody (Affinity, #AF7014) and β-actin (ABclonal, AC026). Following 3 washes in TBST, the membrane was incubated with HRP-inked anti-rabbit IgG secondary antibody (Cell Signaling Technology, 7074) or HRP-linked anti-mouse IgG secondary antibody (CST, 7076S) at room temperature for 1 h. After washing 3 times with TBST, the protein bands were detected with Clarity™ ECL western blot substrate (Bio-Rad, 1,705,060) and visualized using the ChemiDoc Touch imaging system (Bio-Rad).

### Enzyme-Linked Immunosorbent Assay (ELISA)

The levels of TNF-α and IL-6 in the serum and colon supernatants were detected using mouse TNF-α and IL-6 ELISA Ready-SET-Go! Kit (eBioscience, USA), according to the manufacturer’s recommendations. Cytokine concentrations were calculated using the standard curves.

### Bacterial Quantification in Feces

For quantification of total fecal bacterial load, total DNA was isolated from known amounts of feces using the QIAamp DNA Stool Mini Kit (Qiagen). DNA was then subjected to quantitative PCR using the QuantiFast SYBR Green PCR Kit (Biorad) with universal 16S rRNA primers (5’-AGAGTTTGATCCTGGCTCAG-3′ and 5′-CTGCTGCCTCCCGTAGGAGT-3′) to measure total bacteria number. Results are expressed as bacteria number per mg of stool, using a standard curve.

### Statistical Analysis

Data were analyzed using GraphPad Prism software 8.0 and are presented as the mean ± standard error of mean (SEM). After data were tested for normality, the differences among the intervention groups were determined using one-way analysis of variance (ANOVA) followed by the *post hoc* Tukey-Kramer test. A *P* values < 0.05 were considered to indicate statistical significance. For 16S rRNA gene sequencing analysis, statistical analyses were conducted with R software (version 2.15.3), and functional differences in orthologs among groups were assessed by a one-way ANOVA followed by *post hoc* Tukey-Kramer test and wilcox test for multiple comparisons. The predicted functional composition of the intestinal microbiome was inferred for each sample using Phylogenetic Investigation of Communities by Reconstruction of Unobserved States (PICRUSt) based on the Kyoto Encyclopedia of Genes and Genomes (KEGG) functional pathway. Statistical analyses were conducted with Statistical Analysis of Metagenomic Profiles (STAMP), and functional differences in orthologs among groups were assessed by a one-way ANOVA followed by *post hoc* Tukey-Kramer multiple comparisons. The presence of outliers was assessed using the Grubbs test. Handling of data was performed in a blinded manner in accordance with recent recommendations from a National Institutes of Health (NIH) Workshop on preclinical models of neurological diseases (46).

## Results

### ESPs Supplementation Ameliorates Cognitive Impairment in HF Diet-Fed Mice

**Figure 1:**
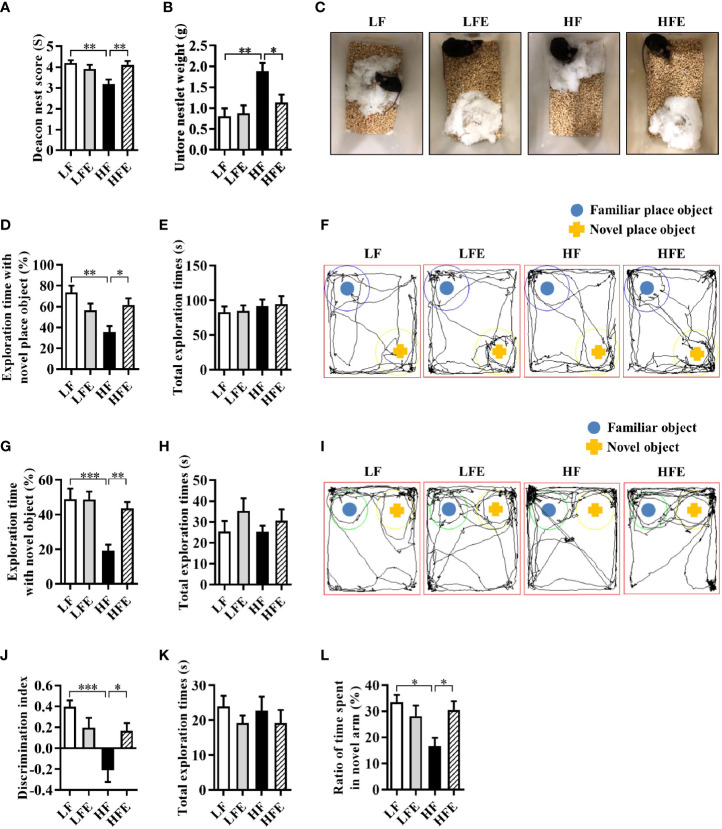
ESPs supplementation prevented cognitive impairment in HF diet-induced obese mice. The nest building test was used to assess the activities of daily living of mice **(A–C)**. **(A)** The nest score and **(B)** untore nestlet weight (amount of untore nesting material). **(C)** Representative nest results of the low-fat (LF), ESPs supplementation in LF (LFE), high fat (HF), and ESP supplementation in HF (HFE) groups. The object location test was performed to evaluate the spatial memory of mice **(D–F)**. **(D)** Percentage of time spent with the object in the novel place to the total object exploration time. **(E)** The total object exploration time. **(F)** Representative track plots of the LF, LFE, HF, and HFE groups recorded by the SMART video tracking system in the testing phase. Note that the LF mice spent more time exploring the object in the novel place than the HF mice, which did not show any preference to the object in the novel place. The novel object recognition test was performed to evaluate the object recognition memory of mice **(G–I)**. **(G)** Percentage of time spent with the novel object to the total object exploration time. **(H)** The total object exploration time. **(I)** Representative track plots of the LF, LFE, HF, and HFE groups. The temporal order memory test was performed to evaluate the recognition memory of mice **(J, K)**. **(J)** Percentage of time spent with the old familiar object to the total object exploration time. **(K)** The total object exploration time. The Y-maze memory test was performed to evaluate the spatial memory of mice **(L)**. **(L)** Percentage of time spent with the novel arm to the total exploration time. Values are represented as mean ± standard error of the mean (SEM). n = 12. ^*^ *P* < 0.05, ^**^ *P* < 0.01, ^***^ *P* < 0.001. Tukey-Kramer test.

To begin with, we detected the body weight, body fat accumulation and liver weight of mice after chronic HF diet feeding. Compared with the LF group, mice fed a HF diet showed increased body weight, increased body fat accumulation and liver weight (all *P* < 0.01, **Supplementary Figures S2A–E**), which indicated that HF diet-induced obesity model was successfully established. Moreover, there were no significant differences between HF and HFE groups in body weight, body fat accumulation and liver weight (all *P* > 0.05, **Supplementary Figure S2A–E**). Next, to evaluate whether ESPs supplementation could prevent HF diet-induced cognitive impairment, we performed nest building, novel object location, novel object recognition, temporal order memory, and Y-maze memory behavioral tests to explore the ability of animals to perform activities of daily living, recognition memory, and spatial memory. In the nesting behavioral test, mice in the HFE group exhibited higher deacon nest scores (ability to build a nest) than those in the HF group (*P* < 0.01, **Figures 1A, C**). In contrast, the untore nestlet weight (nest-building deficit) of the HFE group was significantly lower than that of the HF group (*P* < 0.05, **Figure 1B**). In the object location test, ESPs supplementation significantly improved the place recognition memory by increasing the place discrimination index (PDI) and percentage of time spent with the object in a novel place compared with HF diet-fed mice (*P* < 0.05, **Figures 1D, F**). The difference in the PDI values of the HF and HFE groups was not due to the variant general activity as all tested groups had similar total exploration time with the objects during the test phases (*P* > 0.05, **Figure 1E**). In the novel object recognition test, the novel object discrimination index (NODI) was significantly decreased in the HF diet-fed mice than the LF diet-fed mice, while ESPs supplementation effectively increased the NODI in the HF diet-fed mice (*P* < 0.01, **Figures 1G, I**). The total exploration time of the objects during the testing phase was comparable among the four groups (*P* > 0.05, **Figure 1H**). In the temporal order memory test, ESPs supplementation significantly increased the percentage of time spent with the old familiar object in the HF diet-fed mice (*P* < 0.05, **Figure 1J**), suggesting enhanced recognition memory, which was decreased by the HF diet. The total time spent on object exploration in the testing phase was comparable among the four groups (*P* > 0.05, **Figure 1K**). In the Y-maze memory test, the ratio of time spent in a novel arm was markedly lower in the HF diet-fed mice than the LF diet-fed mice, indicating the deficits in spatial working memory caused by the HF diet, which could be ameliorated by ESPs supplementation (*P* < 0.05, **Figure 1L**). Together, these results suggest that ESPs supplementation prevents HF diet-induced cognitive decline in mice.

### ESPs Supplementation Suppresses the Activation of Microglia and Astrocytes and Alleviates Neuroinflammation in the HIP and PFC Regions of HF Diet-Fed Mice

**Figure 2:**
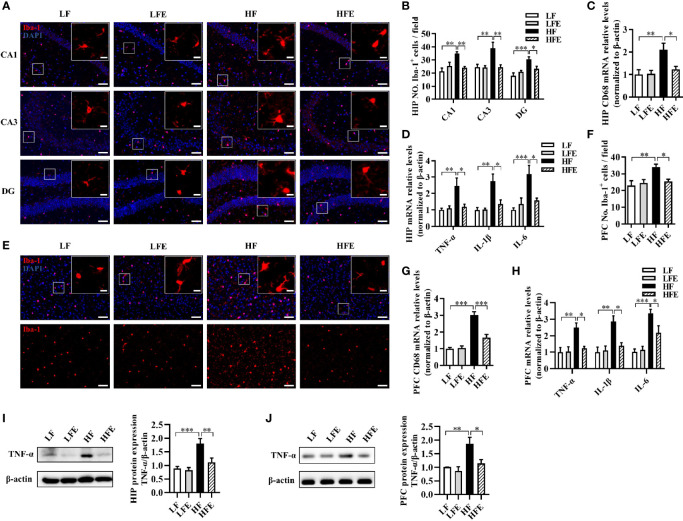
ESPs supplementation suppressed microglial activation and inflammation in the hippocampus and prefrontal cortex of HF diet-induced obese mice. **(A, B)** Representative immunofluorescent staining and quantification of the ionized calcium-binding adaptor molecule 1 (Iba-1^+^) cells in the cornu ammonis 1 (CA1), cornu ammonis 3 (CA3), and dentate gyrus (DG) regions of the hippocampus (HIP) (n = 3, 2 images per mouse, scale bar 50 μm). The image captured from the box was marked with a solid line (scale bar 10 μm). **(C, D)** The mRNA expression levels of the cluster of differentiation 68 (CD68) and proinflammatory cytokines, including the tumor necrosis factor-alpha (TNF-α), interleukin-1 beta (IL-1β), and interleukin-6 (IL-6) in the HIP (n = 6). **(E, F)** Immunofluorescent staining and quantification of Iba-1^+^ cells in the prefrontal cortex (PFC) (n = 3, 2 images per mouse, scale bar 50 μm). **(G, H)** The mRNA expression levels of CD68 and proinflammatory cytokines, including TNF-α, IL-1β, and IL-6 in the PFC (n = 6). The relative mRNA levels were normalized with reference gene (β-actin). **(I, J)** The protein expression levels of TNF-α in the HIP and PFC (n = 8). Values are represented as mean ± SEM. ^*^ *P* < 0.05, ^**^ *P* < 0.01, ^***^ *P* < 0.001. Tukey-Kramer test.

Neuroinflammation induced by the activation of microglia and astrocytes in the HIP and PFC regions is considered to be a critical event that triggers neurodegenerative diseases (2, 6, 7). We determined the effects of ESPs on microgliosis, astrocyte hyperplasia, and neuroinflammation induced by the HF diet. Using the ionized calcium-binding adaptor molecule-1 (Iba-1) as an immunofluorescence marker of microglia, we verified that HF diet increased the number of microglia in the HIP regions, including the cornu ammonis 1 (CA1), cornu ammonis 3 (CA3), and dentate gyrus (DG), while ESPs supplementation significantly decreased the number of microglia in the CA1, CA3, and DG (*P* < 0.05, **Figures 2A, B**). Moreover, mRNA expression of the activated microglial marker, cluster of differentiation 68 (CD68), was significantly higher in the HIP of HF diet-fed mice than the LF and HFE groups (*P* < 0.05, **Figure 2C**). Additionally, ESPs supplementation significantly prevented the upregulation of the expression of proinflammatory cytokines [tumor necrosis factor-alpha (TNF-α), interleukin 1-beta (IL-1β), and interleukin 6 (IL-6)] (all *P* < 0.05, **Figure 2D**). The number of microglia was also increased in the PFC region (*P* < 0.01, **Figures 2E, F**), accompanied by the upregulated expression of CD68 and proinflammatory cytokines in the HF group (all *P* < 0.01, **Figures 2G, H**). However, ESPs supplementation could reverse these changes (all *P* < 0.05, **Figures 2E–H**). Accordingly, ESPs supplementation significantly decreased the protein levels of TNF-α in the HIP and PFC compared to the HF group (all *P* < 0.05, **Figures 2I, J**). Furthermore, using the glial fibrillary acidic protein (GFAP) as a marker of astrocytes, an elevated number of astrocytes was observed in the CA1, CA3, and DG regions of HIP and PFC in HF diet-fed mice (all *P* < 0.001, **Supplementary Figures S3A–D**). Notably, ESPs supplementation alleviated the hyperplasia of astrocytes (all *P* < 0.05, **Supplementary Figures S3A–D**). These results indicate that ESPs prevent the HF diet-induced activation of microglia and astrocytes and alleviate neuroinflammation in the HIP and PFC regions.

### ESPs Supplementation Mitigates Synaptic Impairment in the HIP and PFC of HF Diet-Fed Mice

**Figure 3:**
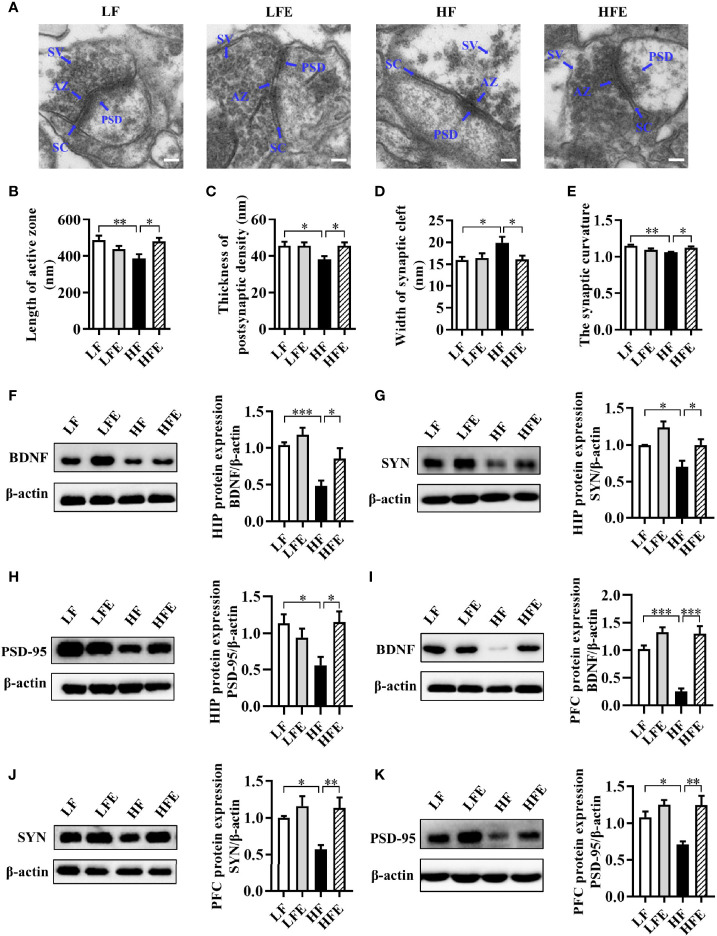
ESPs supplementation ameliorated synaptic impairment in the HIP and PFC of HF diet-induced obese mice. **(A)** Representative ultrastructure of synapses in the CA1 region of mice on the electron micrograph (scale bar 100 nm). **(B–E)** Image analysis of the length of active zone, thickness of postsynaptic density, width of the synaptic cleft, and the synaptic curvature (n = 3, 6 images per mouse). AZ, active zone; PSD, postsynaptic density; SC, synaptic cleft; SV, synaptic vesicle. **(F–H)** The protein expression levels of BDNF, SYN and PSD-95 in the HIP (n = 8). **(I–K)** The protein expression levels of BDNF, SYN and PSD-95 in the PFC (n = 8). Values are represented as mean ± SEM. ^*^ *P* < 0.05, ^**^ *P* < 0.01, ^***^ *P* < 0.001. Tukey-Kramer test.

Following our finding that ESPs prevent neuroinflammation induced by an HF diet, we further evaluated the synaptic ultrastructure of CA1 region of mice. Using transmission electron microscopy, the synaptic ultrastructure of the CA1 region of HIP was analyzed after ESPs consumption. We found that HF diet shortened the length of the active zone (LF: 487.8 ± 24.62; HF: 386.9 ± 23.59), decreased the thickness of the postsynaptic density (LF: 45.62 ± 2.102; HF: 38.03 ± 1.825), broadened the synaptic cleft (LF: 15.59 ± 0.7303; HF: 19.86 ± 1.362), and reduced the curvature of the synaptic interface (LF: 1.152 ± 0.01407; HF: 1.061 ± 0.007393) (all *P* < 0.05, **Figure 3A–E**). However, compared with the HF group, ESPs administration attenuated these synaptic ultrastructure alterations, exhibiting a longer active zone (HFE: 481.8 ± 18.00), thicker PSD (HFE: 46.32 ± 1.948), narrower synaptic cleft (HFE: 16.05 ± 0.8580), and increased synaptic curvature (HFE: 1.123 ± 0.01592) (all *P* < 0.05, **Figures 3A–E**). Meanwhile, we also measured the protein levels of synapse plasticity markers, brain derived neurotrophic factor (BDNF), synaptophysin (SYN) and postsynaptic density-95 (PSD-95) in the HIP and PFC of mice. In the HIP and PFC, long-term HF diet decreased the protein levels of BDNF, SYN and PSD-95 (all *P* < 0.05, **Figures 3F–K**). However, ESPs supplementation significantly attenuated the decline of BDNF, SYN and PSD95 protein levels compared to the HF group (all *P* < 0.05, **Figures 3F–K**). In summary, these results indicate that ESPs improve the synaptic morphology of HIP and PFC, thereby preventing cognitive impairment induced by the HF diet.

### ESPs Supplementation Prevents Hyper-Endotoxemia, Colonic Mucosa Barrier Impairment, and Inflammation in HF Diet-Fed Mice

**Figure 4:**
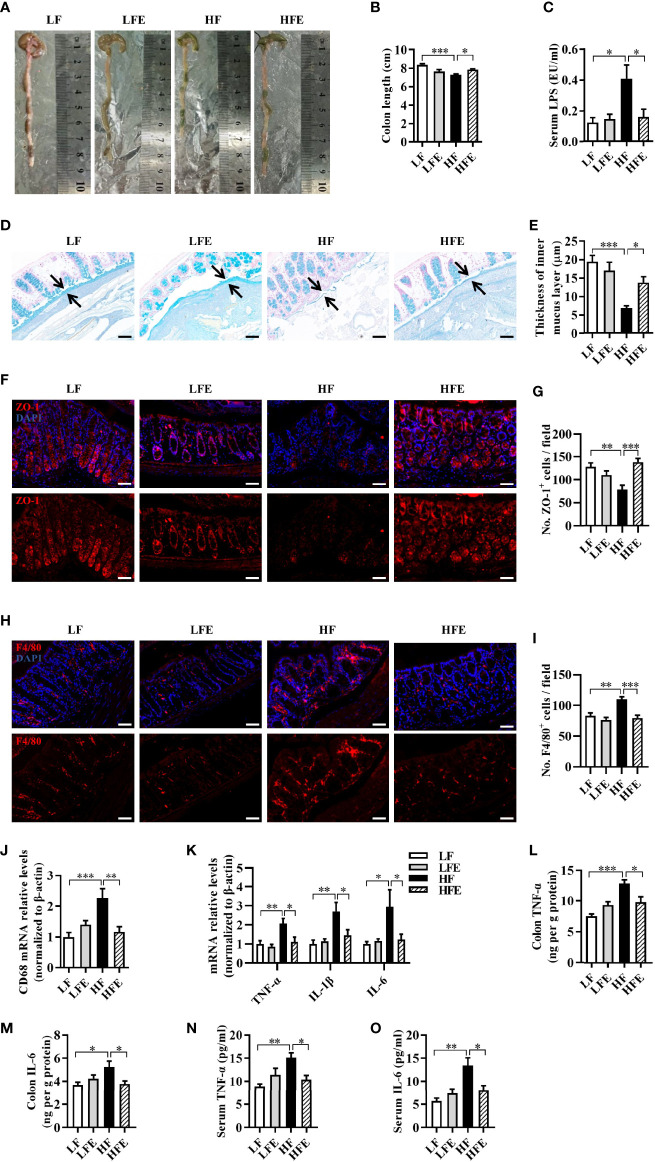
ESPs supplementation prevented mucosa barrier impairment and inflammation in the colon of HF diet-induced obese mice. **(A)** Representative images of the colon. **(B)** Statistical results of colonic length (n = 12). **(C)** Level of lipopolysaccharides (LPS) in the serum. **(D)** Representative images of the Alcian blue-stained colonic sections (scale bar 50 μm), showing the mucus layer (arrows). Opposing black arrows with shafts delineate the mucus layer measured. **(E)** Quantification of the colonic mucus layer was statistically analyzed (n = 3, 2 images per section, 2 sections per mouse). **(F)** Representative immunofluorescence images of the colonic sections stained with the zonula occludens-1 (ZO-1) antibody and 4’, 6-diamidino-2-phenylindole (DAPI). **(G)** Statistical results of ZO-1 positive cells/field (n = 3, 2 images per mouse, scale bar 50 μm). **(H)** Representative immunofluorescence images of the colonic sections stained with the F4/80 antibody and DAPI. **(I)** Statistical results of ZO-1 positive cells/field (n = 3, 2 images per mouse, scale bar 50 μm). **(J, K)** The mRNA expression levels of CD68 **(J)** and proinflammatory cytokines **(K)** in the colon of mice (n = 5-6). The relative mRNA levels were normalized with reference gene (β-actin). **(L–O)** The levels of TNF-α and IL-6 in the serum and colon (n = 6). Values are represented as mean ± SEM. ^*^ *P* < 0.05, ^**^ *P* < 0.01, ^***^ *P* < 0.001. Tukey-Kramer test.

Following our findings that ESPs alleviated neuroinflammation and improved the synaptic morphology, we further investigated the effect of ESPs on the integrity of the gut barrier. We found that ESPs increased the length of the colon compared to that of the HF group (*P* < 0.05, **Figures 4A, B**). Using alcian blue staining to assess the colonic mucus layer, we found that ESPs supplementation significantly increased the thickness of colonic mucus compared with that in HF diet-fed mice (*P* < 0.05, **Figures 4D, E**). Moreover, immunofluorescence staining showed that ESPs also increased the expression of the tight junction protein, zonula occludens-1 (ZO-1), compared with that in HF diet-fed mice (*P* < 0.001, **Figures 4F, G**). Aligning with the improved colonic barrier integrity, ESPs supplementation lowered the LPS concentration in the sera of HF diet-fed mice (*P* < 0.05, **Figure 4C**), which indicated that ESPs attenuated the permeability of the gut to endotoxins. Thus, these results suggest that ESPs alleviate the colonic mucosa barrier impairment induced by the HF diet.

As one of the most abundant immune cells, macrophage plays a key role in intestinal immunity and homeostasis (47, 48). Using the macrophage-specific antigen (F4/80) as a marker of macrophages, we observed that the number of macrophages was significantly increased in the colon of the HF group than the LF group (*P* < 0.01, **Figures 4H, I**), while ESPs effectively prevented the accumulation of macrophages (*P* < 0.001, **Figures 4H, I**). Moreover, ESPs supplementation prevented the upregulation of CD68 mRNA expression induced by the HF diet in the colon (*P* < 0.01, **Figure 4J**), suggesting an improvement in the HF-induced activation of macrophages in the colon. Furthermore, ESPs administration prevented the HF diet induced effects by increasing the mRNA levels of the proinflammatory cytokines, including TNF-α, IL-1β, and IL-6 in the colon (all *P* < 0.05, **Figure 4K**). Accordingly, the increased levels of TNF-α, IL-6 in the colon and serum induced by the HF diet were decreased after ESPs supplementation (all *P* < 0.05, **Figures 4L–O**). The results described above clearly indicate that ESPs supplementation alleviates HF-induced inflammation in the colon and serum. Collectively, these results suggest that ESPs supplementation prevents the colonic mucosa barrier impairment and ameliorates systemic inflammation in the HF diet-induced obese mice.

### ESPs Supplementation Attenuates Gut Microbiota Dysbiosis in HF Diet-Fed Mice

**Figure 5:**
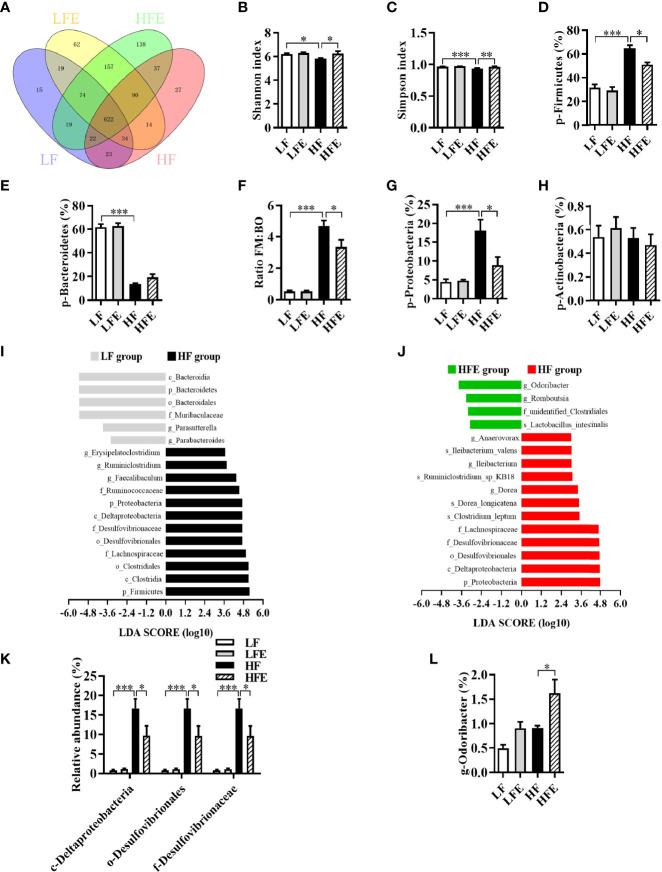
ESPs supplementation attenuated gut microbiota dysbiosis in HF diet-induced obese mice. **(A)** Venn diagram of operational taxonomic units (OTUs). **(B)** Shannon index. **(C)** Simpson index. **(D)** Relative abundance of Firmicutes. **(E)** Relative abundance of Bacteroidetes. **(F)** Ratio of Firmicutes (FM) proportion to Bacteroidetes (BM) proportion (Ratio FM/BO). **(G)** Relative abundance of Proteobacteria. **(H)** Relative abundance of Actinobacteria. **(I, J)** Linear discriminant analysis (LDA) effect size (LEfSe) showing the most differentially significant abundant taxa enriched in microbiota from the HF *vs*. LF and HFE *vs*. HF groups. **(K)** Comparison of the representative taxonomic abundance of Proteobacteria among the LF, LFE, HF, and HFE groups at the class, order and family levels. **(L)** Comparison of the representative taxonomic abundance of Bacteroidetes among the LF, LFE, HF, and HFE groups at the genus level. Values are represented as mean ± SEM. n = 6. ^*^ *P* < 0.05, ^**^ *P* < 0.01, ^***^ *P* < 0.001. LDA score > 3.0 was considered significant. p, phylum; c, class; o, order; f, family; g, genus.

Our previous studies demonstrated that the gut microbiota act as a vital link between the gut pathology and cognitive impairment in obese mice (10, 41, 42). To investigate the effects of HF diet, with or without ESPs supplementation, on gut microbiota, 16S rRNA gene sequencing was used to evaluate the effects of ESPs on the richness, diversity, and composition of gut microbiota. The average operational taxonomic units (OTUs) for each group and overlaps were derived using Venn diagrams. As shown in **Figure 5A**, 622 OTUs were shared among the four groups. Moreover, in the Venn diagrams of the LF and HF groups, 127 unique OTUs were observed in the LF group, 168 unique OTUs were observed in the HF group, and 701 OTUs were shared by both groups (**Supplementary Figure S4A**). In the Venn diagrams of the HF and HFE groups, 98 unique OTUs were present in the HF group, 388 unique OTUs were present in the HFE group, and 771 OTUs were shared by both groups (**Supplementary Figure S4B**). The analysis indicated that the microbiota composition of the LF group was significantly different from that of the HF group, and between the HF group and the HFE group.

Meanwhile, compared with the LF group, the Shannon and Simpson indices of HF group were significantly reduced (all *P* < 0.05, **Figures 5B, C**), while ESPs supplementation significantly increased the Shannon and Simpson indices of HF diet-fed mice (all *P* < 0.05, **Figures 5B, C**). Box plot of weighted Unifrac distance showed that the β-diversity index between LF and HF groups was significant different (*P* < 0.01, **Supplementary Figure S4C**). However, there was no significant difference between HF and HFE groups in β-diversity index (*P* > 0.05, **Supplementary Figure S4C**). At the phylum level, a significant increase in the mean proportion of Firmicutes and a significant decline in the mean proportion of Bacteroidetes were observed in HF diet-fed mice compared to the LF diet-fed mice (all *P* < 0.001, **Figures 5D, E**). Moreover, the ratio of Firmicutes to Bacteroidetes was significantly elevated in the HF group (*P* < 0.001, **Figure 5F**). Importantly, ESPs supplementation prevented the alterations mentioned above (all *p* < 0.05, **Figures 5D, F**), except for the mean proportion of Bacteroidetes (*P* > 0.05, **Figure 5E**). In addition, ESPs significantly decreased the mean proportion of Proteobacteria in HF diet-fed mice (*P* < 0.05, **Figure 5G**). However, there was no significant difference in the abundance of Actinobacteria among the four groups (*P* > 0.05, **Figure 5H**).

Linear discriminant analysis (LDA) effect size (LEfSe) revealed that bacteria belonging to the phyla, Firmicutes and Proteobacteria, as well as those belonging to the genera, *Dubosiella* and *Odoribacter*, were differentially enriched in the gut bacterial communities (LDA score > 3) of the HF and HFE groups (**Figures 5I, J**). Meanwhile, ESPs supplementation decreased the relative abundance of bacteria belonging to the phylum, Proteobacteria (**Figure 5K**), while increasing the relative abundance of genus, *Odoribacter* (**Figure 5L**), short-chain fatty acid (SCFA)-producing bacteria belonging to the phylum, Bacteroidetes. Further analysis revealed that among the four groups, 31 enrichment scores were different in the functionally enriched Kyoto Encyclopedia of Genes and Genomes (KEGG) pathways at level two within 7 seven-level one in the microbiota community (**Supplementary Table S1**). In total, 30 functional orthologs were significantly altered in the two KEGG pathways in the HF group relative to the LF group. Notably, ESPs supplementation was associated with six marked microbial functional shifts in the HF group, including membrane transport, energy metabolism, glycan biosynthesis and metabolism, genetic information processing, folding, sorting and degradation, and circulatory system (**Supplementary Table S1**). Overall, these results suggest that ESPs supplementation attenuates gut microbiota dysbiosis induced by the HF diet.

### Ablation of Gut Microbiota With Antibiotics Eliminates the Beneficial Effects of ESPs Supplementation on the Gut-Brain Axis

Using pearson's correlation analysis to obtain the relationship between the phyla, Firmicutes and Proteobacteria and its lower taxa, cognitive behavior, and serum LPS levels, we observed that the improved microbiota-gut-brain axis may contribute to the protective effects of ESPs on the cognitive functions in obese mice (**Supplementary Figure S5**). To confirm the essential roles of gut microbiota in the beneficial effects of ESPs on cognitive deficits, a cocktail of oral antibiotics (Ab) was used to eliminate the ESPs-induced gut microbiota effects. We found that the long-term ESPs supplement group with a cocktail of oral antibiotics showed a 20-fold reduction in fecal bacterial load (*P* < 0.05, **Supplementary Figure S6**). Then, we investigated the effects of Ab on the cognition of mice in LF diet-fed mice by performing behavioral tests. There were no significant differences between LF and LF+Ab group in the nest behavior, object location, novel object recognition, temporal order memory and Y-maze memory tests (all *P* > 0.05, **Supplementary Figure S7A–D**). Also, we evaluated the effects of Ab on the cognitive function of mice under obesity condition (**Supplementary Figure S8**). Compared with the LF group, HF and HF+Ab group showed poorer cognition index (all *P* < 0.05, **Supplementary Figure S8A–C, E**). Importantly, there were no significant differences between HF and HF+Ab groups in the nesting behavior, object location, novel object recognition and Y-maze memory tests (all *P* > 0.05, **Supplementary Figure S8A–C, E**). However, in the temporal order memory test, HF+Ab group showed higher discrimination index than HF group (*P* < 0.05, **Supplementary Figure S8D**). Overall, there results indicate that Ab have little effect on the cognition of mice in this study.

**Figure 6:**
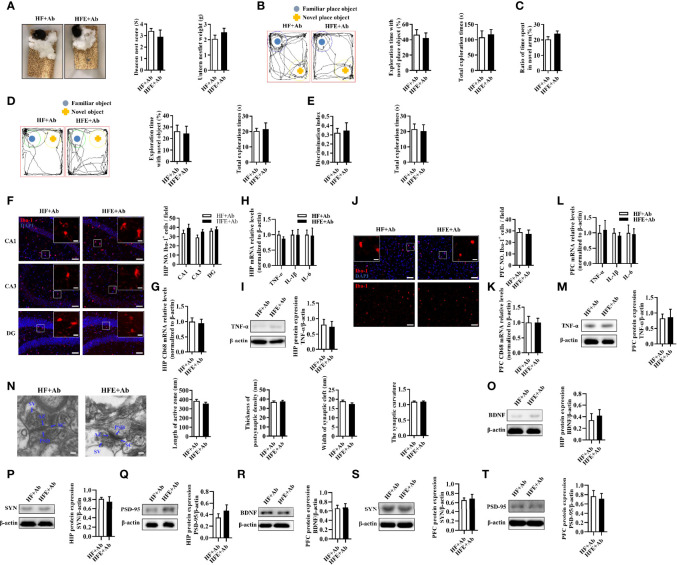
Microbiota ablation with antibiotics eliminated the protective effects of ESPs on cognitive impairment, neuroinflammation, and synapse in HF diet-induced obese mice. **(A)** The nesting behavior test (n =12). **(B)** The object location test (n =12). **(C)** The Y-maze memory test (n =12). **(D)** The novel object recognition test (n =12). **(E)** The temporal order memory test (n =12). **(F)** Representative immunofluorescent staining and quantification of Iba-1^+^ cells in the CA1, CA3, and DG regions of HIP (n = 3, 2 images per mouse, scale bar 50 μm). The image captured from the box was marked with a solid line (scale bar 10 μm). **(G, H)** The mRNA expression levels of CD68 and proinflammatory cytokines, including TNF-α, IL-1β, and IL-6 in the HIP (n = 6). The relative mRNA levels were normalized with reference gene (β-actin). **(I)** The protein expression levels of TNF-α in the HIP (n = 8). **(J)** Immunofluorescent staining and quantification of Iba-1^+^ cells in the PFC (n = 3, 2 images per mouse, scale bar 50 μm). **(K, L)** The mRNA expression levels of CD68 and proinflammatory cytokines, including TNF-α, IL-1β, and IL-6 in the PFC (n = 6). The relative mRNA levels were normalized with reference gene (β-actin). **(M)** The protein expression levels of TNF-α in the PFC (n = 8). **(N)** Representative ultrastructure of synapses in the CA1 region of mice on the electron micrograph (scale bar 100 nm) and image analysis of the length of active zone, thickness of PSD, width of the synaptic cleft, and the synaptic curvature (n = 3, 6 images per mouse). **(O–Q)** The protein expression levels of BDNF, SYN and PSD-95 in the HIP (n = 8). **(R–T)** The protein expression levels of BDNF, SYN and PSD-95 in the PFC (n = 8). Values are represented as mean ± SEM.

After clarifying the role of antibiotics in cognition in this study, we further explored whether ESPs’ beneficial effect on cognition depends on gut microbiota. Compared with the HF+Ab group, the ESPs supplement group did not show an increased deacon nest score and decreased untore nestlet weight in the nest behavior test (*P* > 0.05, **Figure 6A**). Moreover, there were no significant behavioral differences between the HF+Ab and HFE+Ab groups in the object location, novel object recognition, temporal order memory, and Y-maze memory tests (all *P* > 0.05, **Figures 6B–E**). In addition, staining with Iba-1 and GFAP showed that the effects of ESPs, which prevented the gliosis of microglia and astrocytes and neuroinflammation in the HIP (all *P* > 0.05, **Figures 6F–I** and **Supplementary Figures S9A, B**) and PFC (all *P* > 0.05, **Figures 6J–M** and **Supplementary Figures S9C, D**) regions, completely disappeared. Finally, antibiotics eliminated the ameliorating effects of ESPs on synaptic impairment (all *P* > 0.05, **Figures 6N–T**). Overall, these findings of antibiotic intervention suggest that ESPs’s beneficial effects of improving the cognitive impairment, neuroinflammation, and synaptic impairment were closely related to gut microbiota.

In addition, we evaluated whether ESPs’ protective effect on colon homeostasis relies on the gut microbiota. There were no significant changes in colonic length or colonic mucus thickness between the HF+Ab and HFE+Ab groups (all *P* > 0.05, **Supplementary Figures S10A–D**). Further, they exhibited comparable expression levels of the ZO-1 protein (*P* > 0.05, **Supplementary Figures S10E, F**). Similarly, the serum LPS levels were not significantly different in the HF+Ab and HFE+Ab groups (*P* > 0.05, **Supplementary Figure S10G**). In addition, there was similarity in the number of macrophages and the mRNA expression levels of CD68, IL-6, TNF-α, and IL-1β in the colon between the two groups (all *P* > 0.05, **Supplementary Figures S10H–K**). Accordingly, ELISA results showed that the effects of ESPs to decrease levels of TNF-α and IL-6 in the colon and serum were eliminated (all *P* > 0.05, **Supplementary Figures S10L–O**). These findings indicate that antibiotics eliminate the effects of ESPs supplementation on colonic integrity and inflammation. Notably, gut microbiota may play a critical role in ESPs’s effects.

## Discussion

In the present study, using an obese cognitive impairment mouse model, we demonstrated the beneficial effects of larval *E. granulosus-*derived ESPs on the gut microbiota dysbiosis and improvement of cognitive decline. For the first time, we present evidence that ESPs supplementation significantly improves cognitive impairment in HF diet-fed mice, which was supported by the inhibition of proliferation, activation of microglia and astrocytes, alleviation of neuroinflammation, and improvement of synaptic ultrastructure. Surprisingly, the intraperitoneal administration of ESPs significantly mitigated the impairment of colonic barrier and alleviated inflammation and gut dysbiosis in the HF diet-fed mice. Moreover, using a broad-spectrum antibiotic intervention, the abrogation of ESP-induced improvement in the gut-brain axis highlights the essential role of gut microbiota in mediating cognitive function and behavior.

Parasites have evolved to modulate the host immune and tissue repair responses to promote their survival by limiting inflammation that would otherwise drive their expulsion and cause pathology (21, 30). It is well known that parasitic infection generally induces a type 2 immune response, thereby creating an anti-inflammatory immune microenvironment and improving the host metabolic disorders, including obesity (49). Our research group has focused on the infectious immunity of larval *E. granulosus* for a long time. Previously, we reported the accumulation of immunosuppressive cells in the host following infection with this parasite (32, 34, 35). Moreover, ESPs derived from parasites can inhibit the pro-inflammatory responses *in vitro* (34, 36). Recently, we found that parasitic infection also alters the metabolism and differentiation of adipose tissues in mice (37). In this study, we demonstrated a novel function of the larval *E. granulosus*-derived ESPs in preventing obesity-induced cognitive decline, which extends our understanding of parasite derived ESPs in treating neuroinflammation associated disease. Our previous studies have shown that the ESPs consist of thousands of predicted complex components which may contribute to the metabolic adaptation of parasite in hosts (50). In addition, the ESPs derived from *E. granulosus* PSCs were proved to be heat-labile and carbohydrate-dependent in the manipulation of regulatory B cells and Th17 cells induction *in vitro* (34). Given the complex composition and characteristics of ESPs, it is necessary to further purify the drug candidates with the beneficial effect on cognition impairment from the mixed ESPs in the future.

Neuroinflammation has been indicated to be involved in many neurodegenerative diseases (7). It is increasingly recognized that chronic activation of the microglia and astrocytes can maintain a sustained production of proinflammatory cytokines, thereby contributing to the progression of neurodegenerative diseases (2, 5, 6). Here, we found that a chronic HF diet increased the activation and accumulation of microglia and astrocytes as well as the mRNA expression of proinflammatory cytokines in the HIP and PFC regions, which were later attenuated by ESPs supplementation, indicating that ESPs exhibit anti-neuroinflammatory effects (**Figure 2** and **Supplementary Figure S3**). Synaptic ultrastructural damage and atypical synaptic plasticity are closely associated with neuroinflammation (51). Aligning with the downregulated neuroinflammation, ESPs supplementation effectively reversed the damage of synaptic ultrastructure in the HIP of mice, which explained the improvement of cognitive function in mice, despite being fed a chronic HF diet. In this study, we demonstrated that ESPs administration suppresses neuroinflammation and activation of the microglia and astrocytes by improving gut-brain axis. However, it is still not clear whether ESPs can directly cross the blood-brain barrier and suppress the neuroinflammation in the HIP and PFC.

The gut has been widely considered to be closely linked to neural function *via* the gut-brain axis (12, 13). Previous studies have shown that an HF diet markedly increases intestinal inflammation, while diminishing the intestinal barrier integrity. This may result in the release of bacterial LPS into the bloodstream, thereby leading to endotoxemia and systemic inflammation, which eventually lead to cognitive decline (5, 52). In this study, we observed that ESPs administration increased the thickness of colonic mucus as well as the expression of colonic ZO-1 protein and reduced the level of serum LPS. Consistently, we found less proliferation and activation of macrophages accompanied by the downregulated expression of pro-inflammatory cytokines in the colon of HF diet-fed mice (**Figure 4**), which aligns with the downregulated immunological effects mediated by ESPs in other studies (32, 34, 35). Therefore, it is suggested that ESPs maintain the gut functional homeostasis by modulating the host immune responses.

It is noteworthy that the gut microbiota are key regulators of the host intestinal barrier integrity, inflammation, and endotoxemia and also act as a vital link of the gut-brain axis (53). Many studies, including ours, have concluded that a chronic HF diet induces alterations in the richness, diversity, and composition of the gut microbiota in mice, such as decreased Shannon index and Simpson index and increased representation of bacteria belonging to the phyla, Firmicutes and Proteobacteria (10, 14, 41, 42). In the present study, we found that the abundances of the phyla, Firmicutes and Proteobacteria, were significantly decreased after ESPs supplementation. LDA further revealed that ESPs supplementation not only decreased the phylum, Proteobacteria, but also its lower taxa, such as the class Deltaproteobacteria, order Desulfovibrionales, and the family *Desulfovibrionaceae* in HF diet-fed mice (**Figure 5**). These results suggest that ESPs alleviate the gut microbiota dysbiosis in HF diet-fed mice. Interestingly, we observed that the abundance of SCFA (butyrate)-producing bacteria (genus *Odoribacter*) was significantly elevated in HF diet-fed mice following ESPs supplementation (**Figure 5**). SCFAs play important roles in metabolism, with butyrate acting as a metabolic substrate for colonic epithelial cells (54). In obesity, butyrate supplementation is reported to maintain the intestinal epithelial integrity, thereby preventing the occurrence of HF diet-induced metabolic endotoxemia (54). In the present study, we observed improved gut function and altered microbiota composition in HF diet-fed mice following ESPs supplementation. In a previous study, novel object recognition, but not spatial, memory was impaired in antibiotic-treated mice (55). However, we found that Ab had little effects in cognition in this study (**Supplementary Figure S7, S8**). After clarifying the role of antibiotics in cognition in this study, we further used Ab to ablate gut microbiota to explore the role of gut microbiota in ESPs-induced improvement and found that there were no significant differences in HF+Ab and HFE+Ab groups (**Figure 6** and **Supplementary Figures S9, S10**), which indicated that broad-spectrum antibiotic intervention abrogated effects of ESPs in gut microbiota dysbiosis and cognitive decline. These findings of antibiotic intervention indicated that gut microbiota play an important role in ESPs-induced beneficial effects, rather than antibiotics directly affect the observed phenotypes.

**Figure 7:**
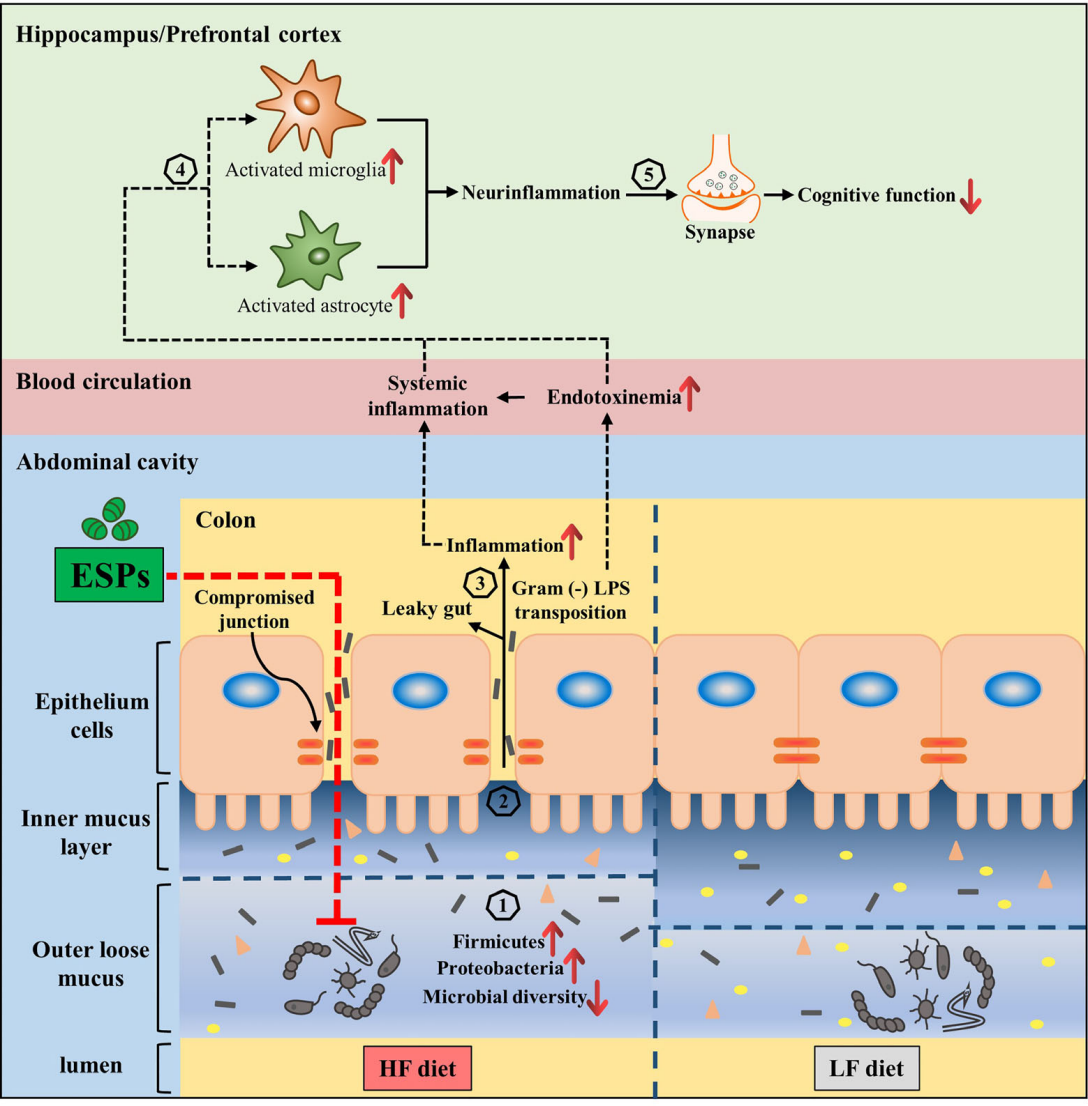
Interplay between the microbiota and gut-brain axis in high-fat diet and ESPs intervention. Gut microbiota regulates the gut-brain axis to maintain good health, while its alteration (increased of Firmicutes and Proteobacteria, decrease of Bacteroidetes and microbial diversity) due to HF diet is related to obesity, which has adverse consequences on cognition (Steps 1–5). ESPs supplementation is thought to decrease Firmicutes and Proteobacteria, while increasing the microbial diversity (1), thereby maintaining the gut mucus and epithelial integrity as well as immune homeostasis (2); this attenuates the translocation of LPS (3), which decreases the peripheral inflammatory tone and inhibits the activation of microglia and astrocytes, causing neuroinflammation (4) and synaptic damage in the central nervous system (5). Therefore, the supplementation of ESPs has a beneficial impact on cognition *via* restoration of the gut microbiota and its regulatory role in the gut-brain axis.

The microbiota-gut-brain axis has been increasingly recognized as a novel target for preventing neurodegenerative diseases (11, 19). In a previous study, we reported that dietary supplementation with various fiber β-glucans can improve the cognitive decline induced by HF diet *via* remodeling of the microbiota composition (10). The present study also demonstrates the neuroprotective function of ESPs, resulting from the alleviation of gut microbiota dysbiosis. Herein, we would like to emphasize that unlike dietary intervention, in this study, ESPs were administered to the HF diet-fed mice *via* intraperitoneal injection. As ESPs administration could decrease the accumulation of macrophages and alleviate HF-induced colonic inflammation (**Figure 4**), we speculated that ESPs maybe indirectly reshape gut microbiota *via* potential anti-inflammatory or immunoregulatory actions that suppress the gut pathology and protect against dysbiosis. In fact, previous studies have showed that several cytokines can influence and shape the microbial community within the gut lumen (56). Moreover, intestinal inflammation can lead to an altered composition of gut microbiota, known as dysbiosis, that is associated with functional changes in the microbial transcriptome, proteome or metabolome (57). Notably, subcutaneous administration of parasitic worm product ES-62 was shown to normalize gut microbiota in collagen-induced arthritis (58). Therefore, maintaining intestinal homeostasis by reducing intestinal inflammation and improving gut pathology may be a feasible way to improve gut microbiota dysbiosis. Thus, the present study proposed that ESPs may act on immune cells to restore gut function, thereby reshaping gut microbiota and improving the microbiota-gut-brain axis (**Figure 7**). However, additional studies are required to identify the potential mechanisms.

There is increasing evidence that parasites or their derived ESPs can be exploited to develop novel therapies for the prevention of inflammatory or metabolic diseases. For example, the parasitic infection by *Trichuris muris* was reported to protect mice deficient in *NOD2* (susceptibility gene for Crohn’s disease) from intestinal abnormalities (59). In addition, the live ova from *Trichuris suis* were used for the treatment of Crohn’s disease and ulcerative colitis (60). In a phase 1b randomized controlled clinical trial, it was demonstrated that the stage three larvae of the human hookworm, *Necator americanus*, could effectively alleviate obesity and its related metabolic syndromes (26). Considering the biosafety issues and low tolerance of the hosts to live parasites, parasite-derived molecules can be used as alternatives for developing parasite-based therapies (58, 61). For example, Lacto-N-fucopentaose III (LNFPIII), a component of soluble egg antigens of *Schistosoma mansoni*, has been found to alleviate hepatosteatosis and insulin resistance in obese mice (29). Notably, a recent study showed that injection of soluble egg derived from *Schistosoma japonicum* improved lipid metabolism in HF diet-induced obese mice (62). To our knowledge, the present study is the first to reveal that the ESPs of larval *E. granulosus* could improve cognitive decline induced by obesity, which broadens the functions of parasitic molecules in neurodegenerative diseases. Collectively, these studies, including ours, propose that parasitic molecules may act as novel targets for the development of therapeutic intervention strategies against human diseases.

## Conclusion

The present study demonstrated that ESPs supplementation prevents cognitive decline, accompanied by improvement of neuroinflammation and synaptic impairment in a chronic HF diet-induced obese mouse model. Furthermore, ESPs supplementation alleviated the HF diet-induced gut dysbiosis as well as degradation of the colonic mucus barrier and tight junctions. Interestingly, the effects of ESPs in improving the gut-brain axis disappeared following microbiota ablation with a broad-spectrum antibiotic, highlighting the vital role of gut microbiota in ESPs-mediated cognitive effects. This study provides the first evidence that supplementation with parasite-derived ESPs improves cognitive impairments and gut microbiota dysbiosis in HF diet-induced obesity. Our findings highlight the important but neglected role of gut microbiota in non-dietary intervention strategies against obesity-induced cognitive impairments and suggest that parasite-derived molecules may act as novel targets for developing drugs against obesity-related neurodegenerative diseases.

## Data Availability Statement

The original contributions presented in the study are included in the article/**Supplementary Material**. Further inquiries can be directed to the corresponding authors.

## Ethics Statement

The animal study was reviewed and approved by Ethics Committee of Xuzhou Medical University (Xuzhou, China, SCXK (Su) 2020-0048).

## Author Contributions

Conceived and designed the experiments: XY, WP, and FS. Performed the experiments: JW, LZ, YL, and JL. Analyzed the data: YZ, WX, and MD. Contributed reagents/materials/analysis tools: WP, XY, TF, WC, and HL. Wrote the manuscript: JW, YZ, WP, and XY. All authors contributed to the article and approved the submitted version.

## Funding

This work was funded by the National Natural Science Foundation of China (No. 81871670, 81800718 and 82002164), the Natural Science Foundation of Jiangsu Province No. BK20201459 and BK20211055), the Jiangsu Shuangchuang Program, the Training Programs of Innovation and Entrepreneurship for College Students in Jiangsu Province (No. 202010313037Z, 202010313007, 202010313035Z and 202010313009), and the Starting Foundation for Talents of Xuzhou Medical University (No. D2018006). The funders had no role in study design, data collection and analysis, decision to publish, or preparation of the manuscript.

## Conflict of Interest

The authors declare that the research was conducted in the absence of any commercial or financial relationships that could be construed as a potential conflict of interest.

## Publisher’s Note

All claims expressed in this article are solely those of the authors and do not necessarily represent those of their affiliated organizations, or those of the publisher, the editors and the reviewers. Any product that may be evaluated in this article, or claim that may be made by its manufacturer, is not guaranteed or endorsed by the publisher.
