# Supplementary material for: Parasite-Derived Excretory-Secretory Products Alleviate Gut Microbiota Dysbiosis and Improve Cognitive Impairment Induced by a High-Fat Diet
Source: Front Immunol. 2021 Oct 20;12:710513. doi: 10.3389/fimmu.2021.710513 (PMC8564115; doi:10.3389/fimmu.2021.710513)
Supplement: Supplementary file 1 [file DataSheet_1.docx]

**Supporting Material**

**
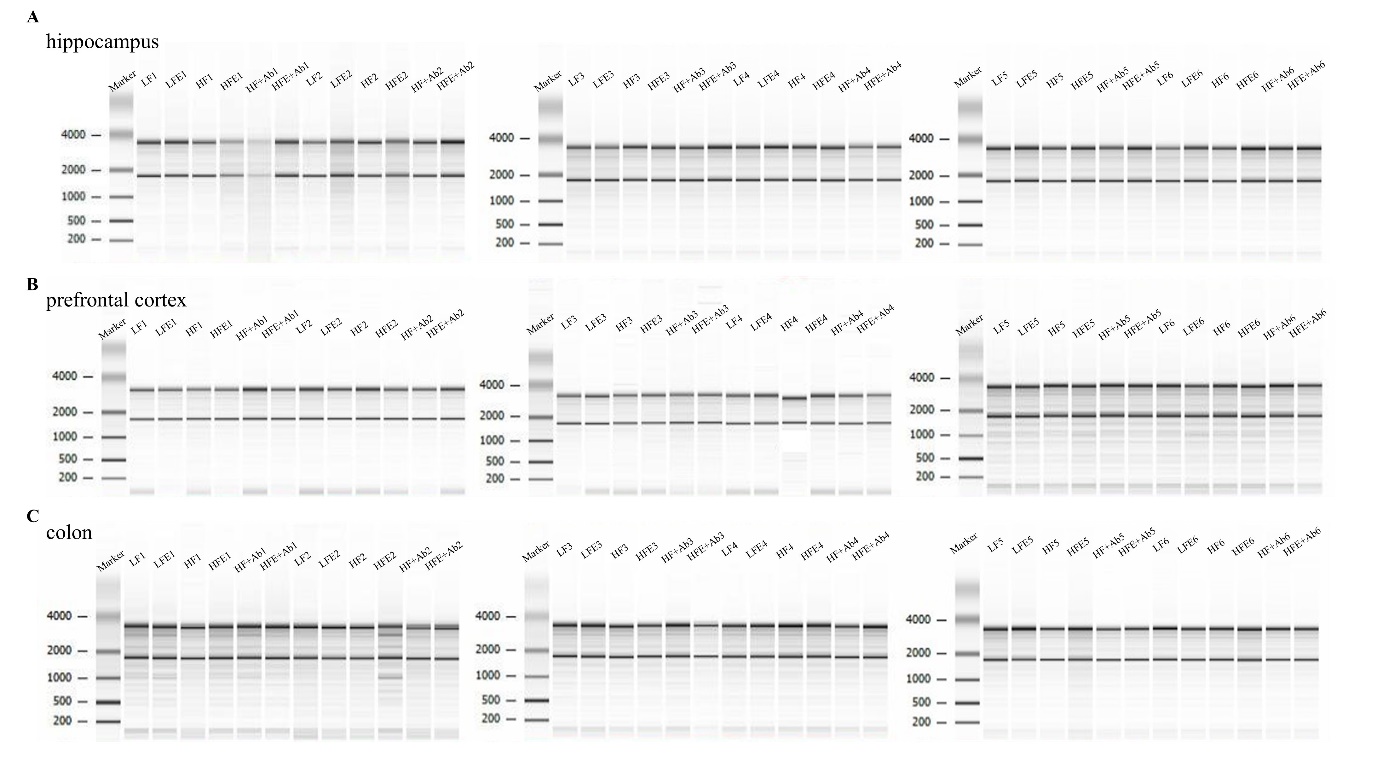
**

**Figure S1. The evidence of tissue RNA integrity in LF, LFE, HF and HFE groups. (A)** Hippocampus. **(B)** Prefrontal cortex. **(C)** Colon.

**
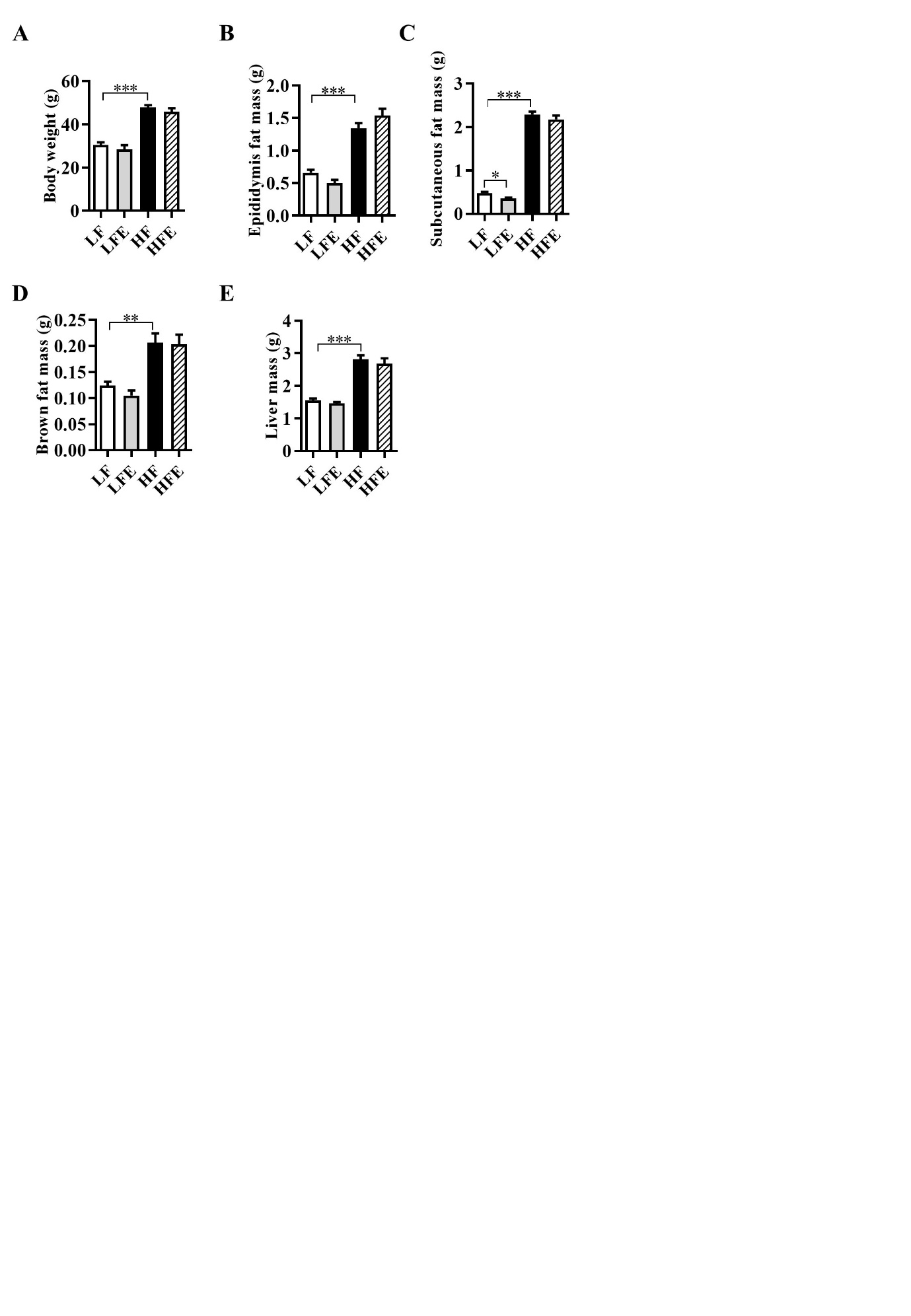
**

**Figure S2. Comparison of the characteristics of the LF, LFE, HF and HFE groups. All measurements for the five characteristic parameters were performed after 15 weeks of diet exposure. (A)** Body weight. **(B-D)** Epididymis, subcutaneous, brown fat mass. **(E)** Liver mass. n=12. Values are mean ± SEM. ^*^*P* < 0.05, ^**^*P* < 0.01, ^***^*P* < 0.001. Tukey-Kramer test.


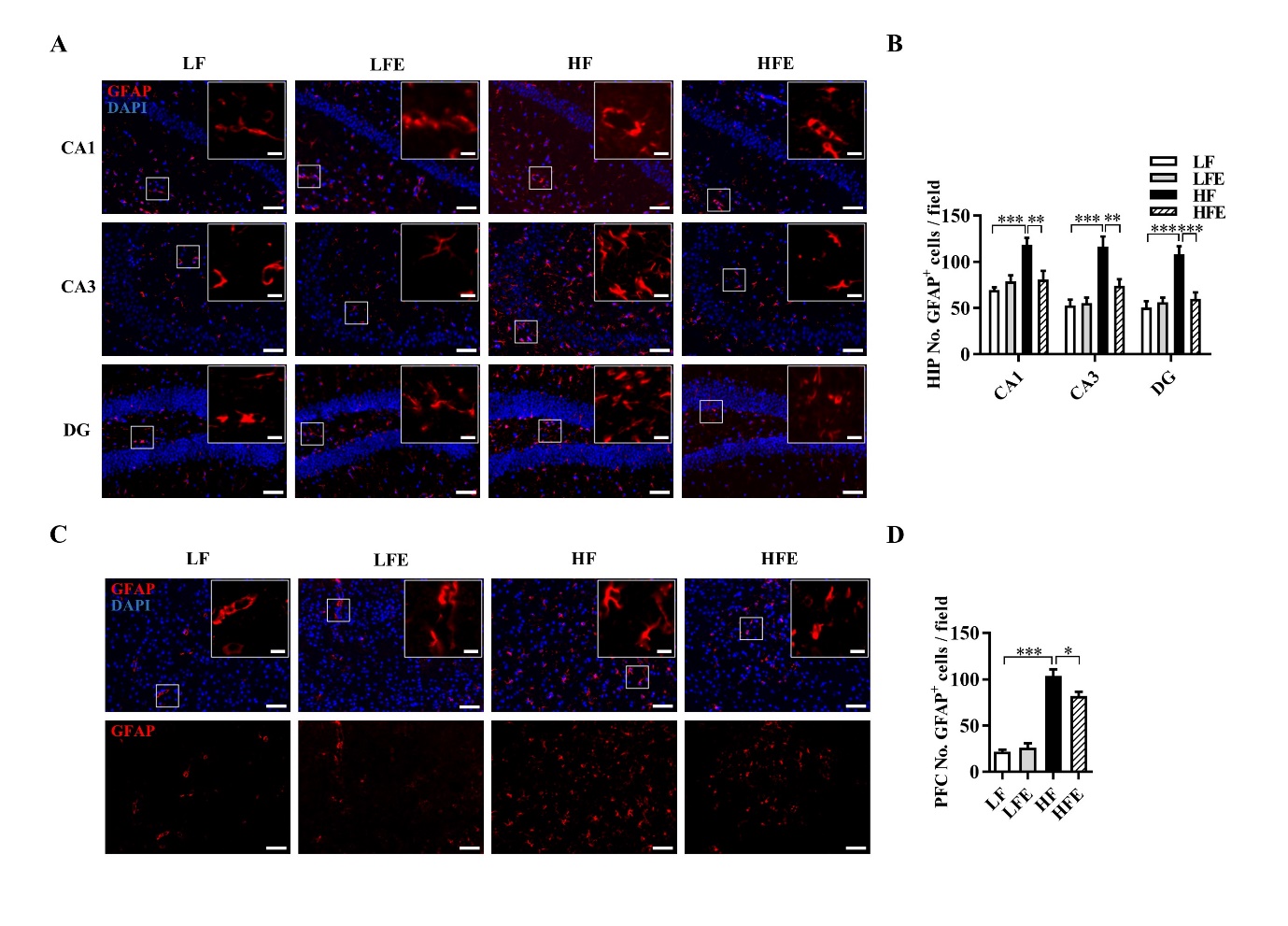


**Figure S3. ESPs supplementation suppressed the activation of astrocytes in the HIP and PFC of HF diet-induced obese mice. (A, B)** The representative immunofluorescent staining and quantification of GFAP^+^ cells numbers in CA1, CA3, and DG regions of the HIP (n= 3, 2 images per mouse, scale bar 50 μm). The image captured from the box was marked with a solid line (scale bar 10 μm). **(C, D)** The immunofluorescent staining and quantification of GFAP^+^ cells numbers in the PFC (n= 3, 2 images per mouse, scale bar 50 μm). Values are mean ± SEM. ^*^*P* < 0.05, ^**^*P* < 0.01, ^***^*P* < 0.001. Tukey-Kramer test.


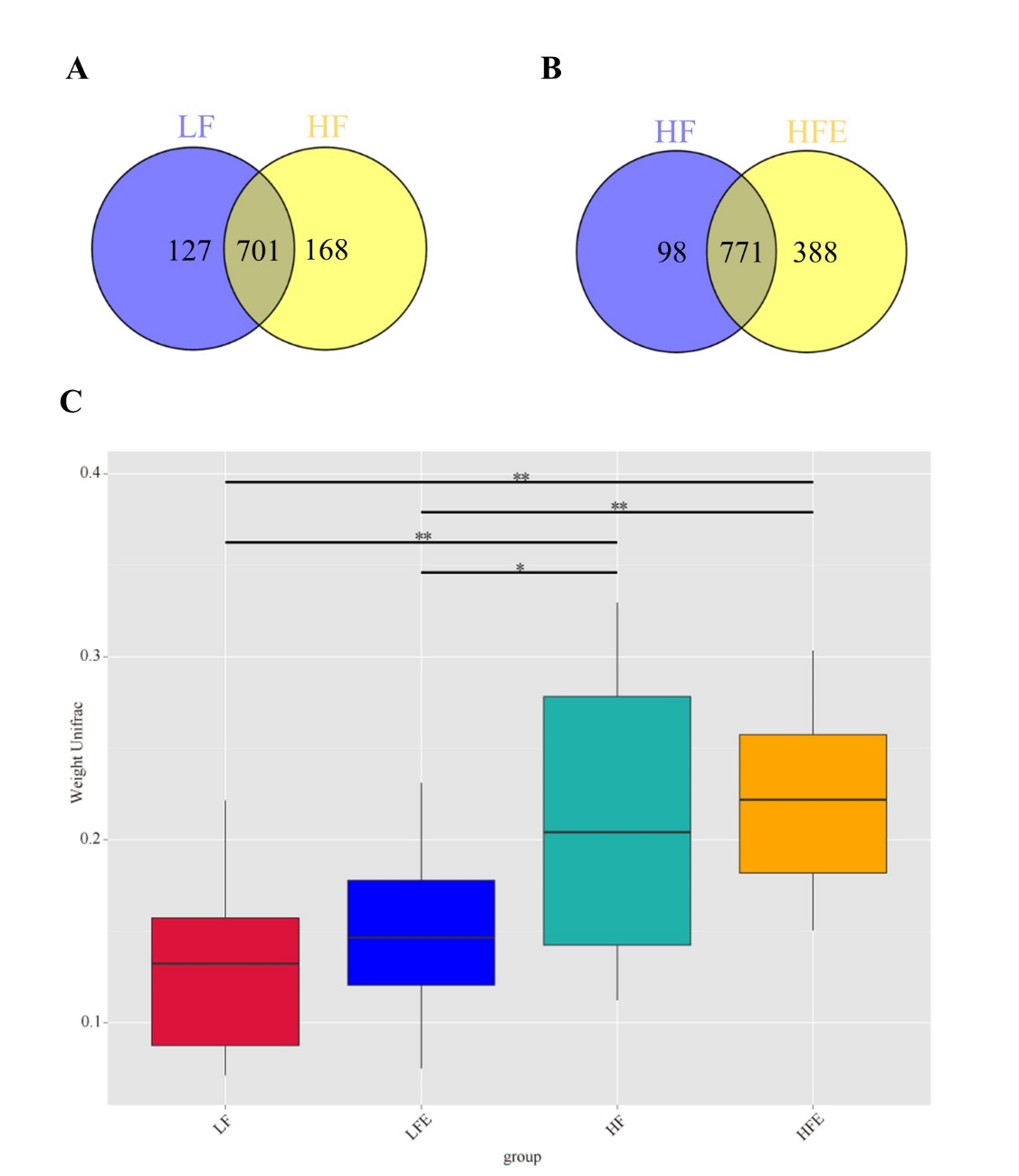


**Figure S4. Effects of ESPs supplementation on the OTUs and β-diversity index of gut microbiota. (A)** Venn diagram between LF and HF groups. **(B)** Venn diagram between LF and HF groups. Sequences with ≥97% similarity were assigned to the same OTUs. **(C)** Box plot of weight Unifrac distance. ^*^*P* < 0.05, ^**^*P* < 0.01. Tukey-Kramer test.


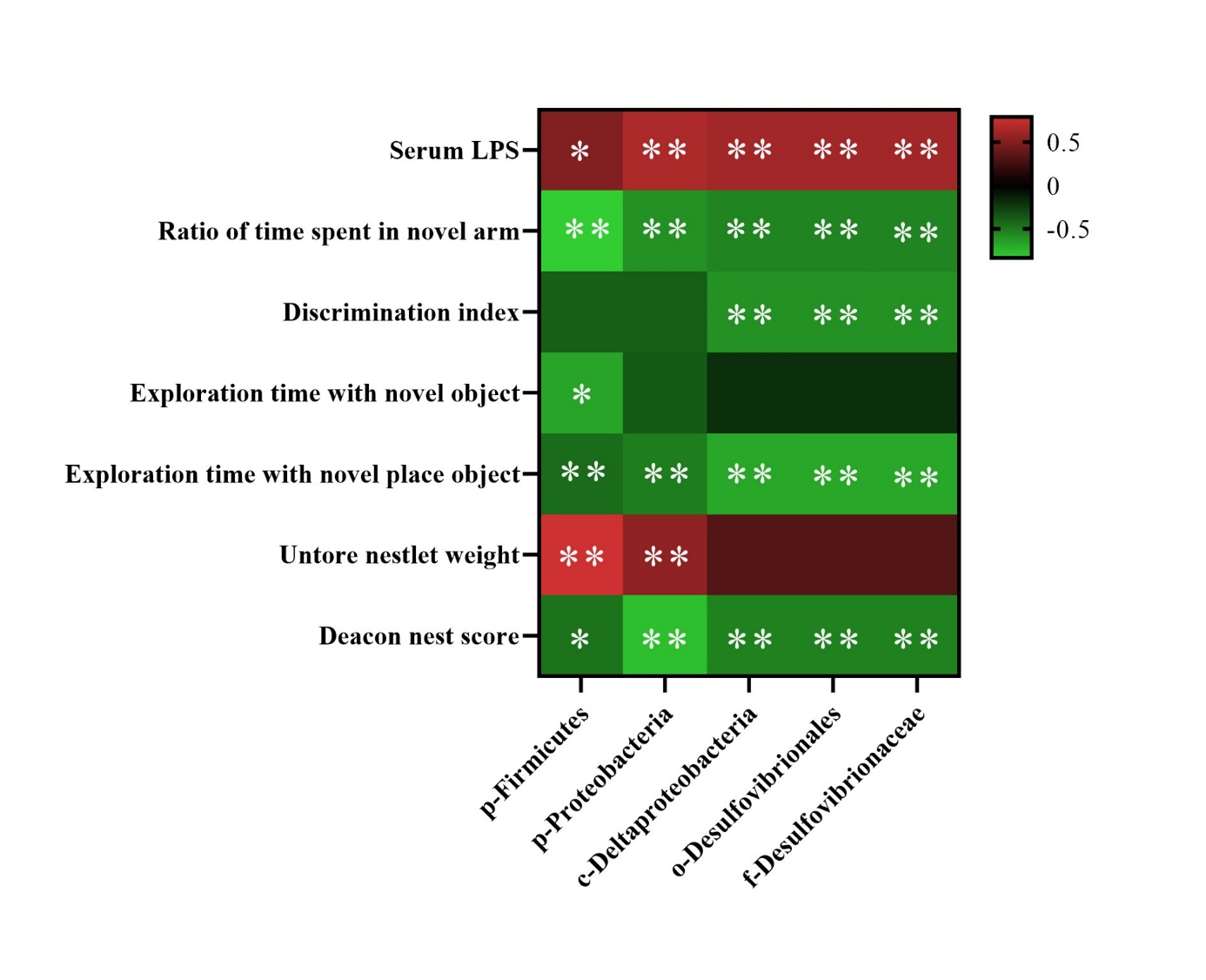
**Figure S5. Pearson’s correlations between *Firmicutes*, *Proteobacteria* and its down taxa, and cognitive behaviour and serum LPS levels.** ^*^*P*< 0.05, ^**^*P* < 0.01.


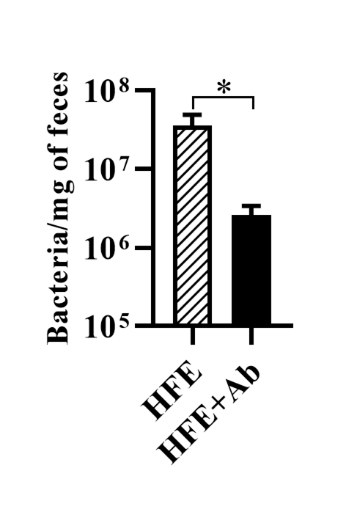


**Figure S6. Antibiotics significantly decreased bacterial DNA of faces in HFE mice (n = 6).** ESPs supplementation in HF (HFE) group vs. ESPs supplement with antibiotics (HFE+AB) group. ^*^*P* < 0.05. Values are mean ± SEM.


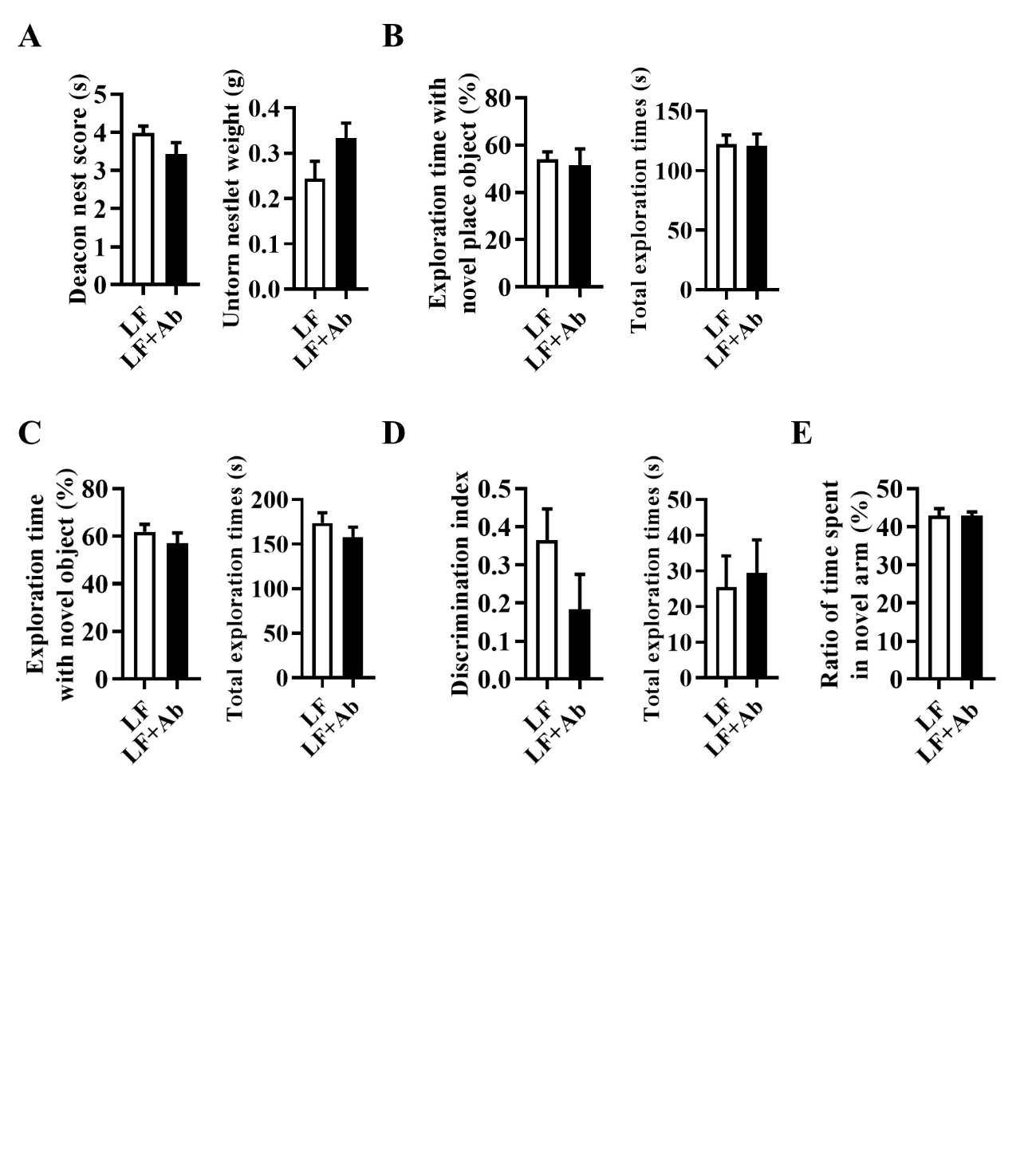


**Figure S7. Effects of antibiotics on the cognitive function of LF diet-fed mice.** 8 weeks old mice were randomly divided into LF and LF+Ab groups and LF+Ab group mice were treated with antibiotics in the drinking water. Behavior tests were performed at the fourth week treatment. **(A)** The nest score and untore nestlet weight (amount of untore nesting material) in the nesting behavior test. **(B)** Percentage of time spent with the object in the novel place to the total object exploration time and total object exploration time in the object location test. **(C)** Percentage of time spent with the novel object to the total object exploration time and the total object exploration time in the novel object recognition test. **(D)** Percentage of time spent with the old familiar object to the total object exploration time and the total object exploration time in the temporal order memory test. **(E)** Percentage of time spent with the novel arm to the total exploration time in the Y-maze memory test. Values are represented as mean ± standard error of the mean (SEM). n = 12. ^*^*P* < 0.05, ^**^*P* < 0.01. Tukey-Kramer test.


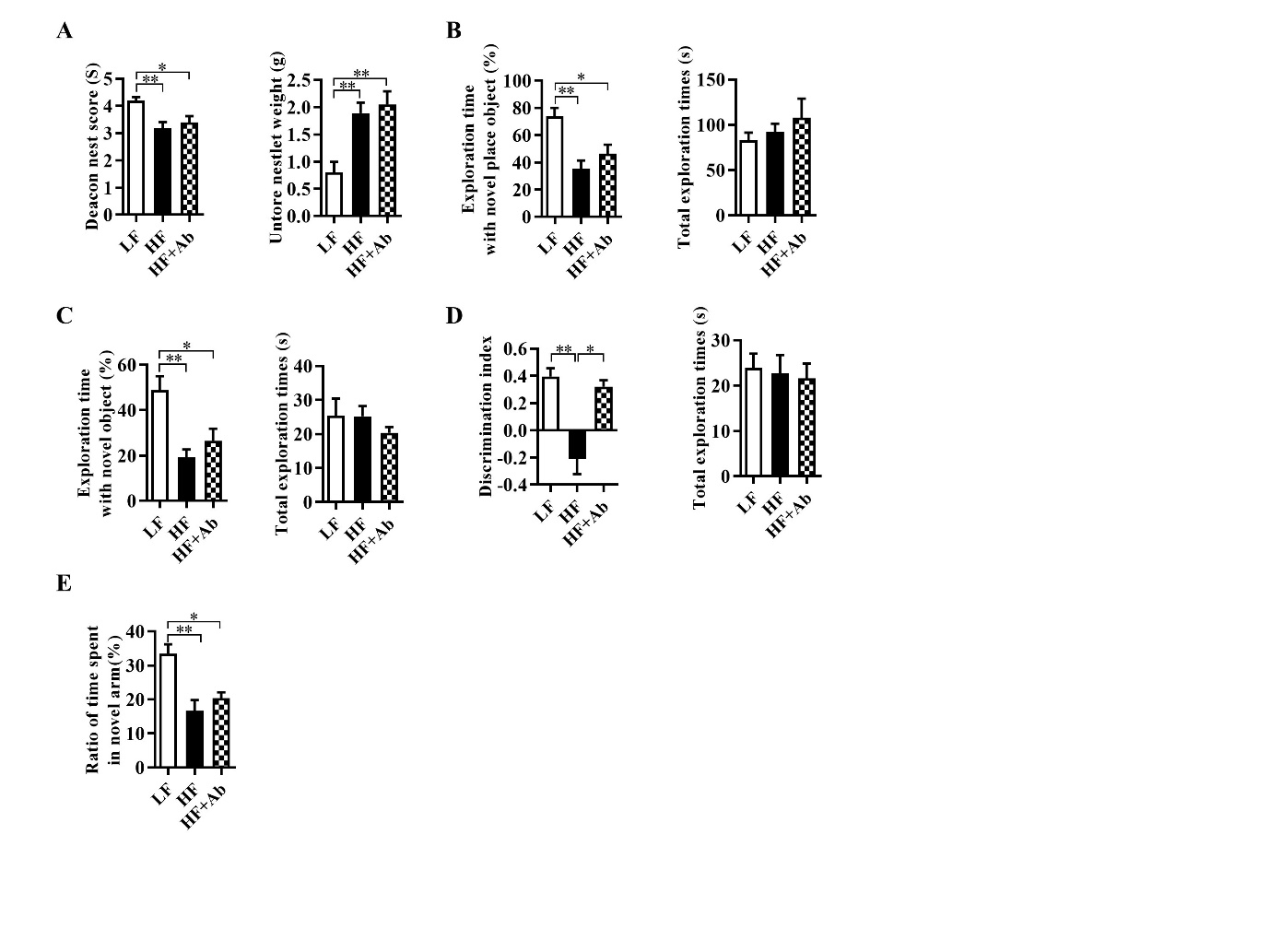


**Figure S8. Effects of antibiotics on the cognitive function of mice under obesity condition.** **(A)** The nest score and untore nestlet weight (amount of untore nesting material) in the nesting behavior test. **(B)** Percentage of time spent with the object in the novel place to the total object exploration time and total object exploration time in the object location test. **(C)** Percentage of time spent with the novel object to the total object exploration time and the total object exploration time in the novel object recognition test. **(D)** Percentage of time spent with the old familiar object to the total object exploration time and the total object exploration time in the temporal order memory test. **(E)** Percentage of time spent with the novel arm to the total exploration time in the Y-maze memory test. Values are represented as mean ± standard error of the mean (SEM). n = 12. ^*^*P* < 0.05, ^**^*P* < 0.01. Tukey-Kramer test.


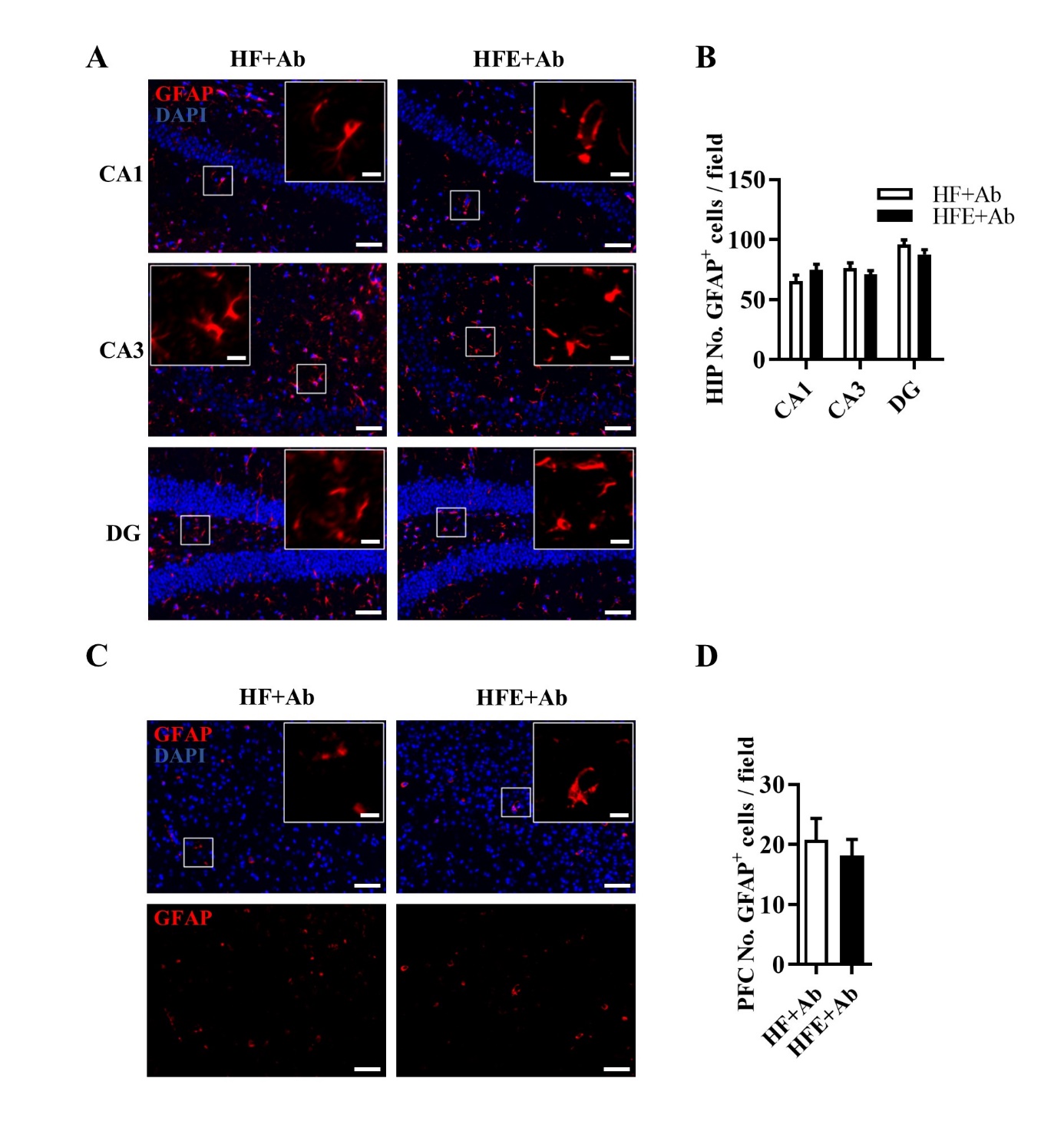


**Figure S9. Microbiota ablation with antibiotics eliminated the effects of ESPs supplementation in suppressing the activation of astrocyte.** **(A, B)** The representative immunofluorescent staining and quantification of GFAP^+^ cells numbers in CA1, CA3, and DG regions of the HIP (n= 3, 2 images per mouse, scale bar 50 μm). The image captured from the box was marked with a solid line (scale bar 10 μm). **(C, D)** The immunofluorescent staining and quantification of GFAP^+^ cells numbers in the PFC (n= 3, 2 images per mouse, scale bar 50 μm). Values are mean ± SEM.


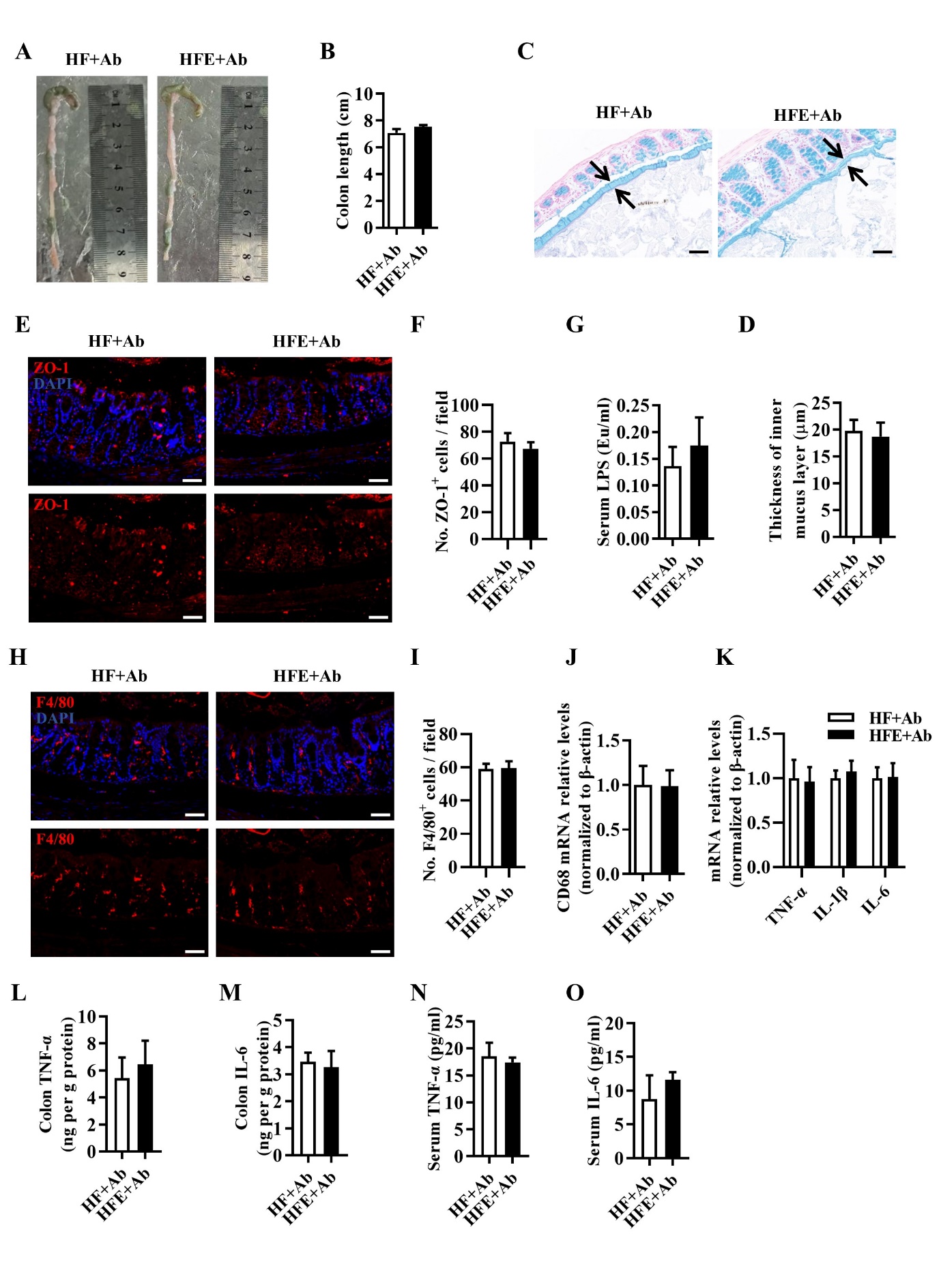


**Figure S10. Microbiota ablation with antibiotics eliminated the effects of ESPs supplementation in preventing the mucosa barrier impairment and inflammation in the colon of HF diet-induced obese mice. (A)** The representative pictures of colon. **(B)** The statistical results of colonic length (n=12). **(C)** The representative pictures of Alcian blue-stained colonic sections, which were showing the mucus layer (arrows). Opposing black arrows with shafts delineate the mucus layer that was measured. **(D)** The quantification of the colonic mucus layer was statistically analyzed (2 sections per animal, 2 images per section, n=3). **(E)** The representative immunofluorescence images of colonic sections stained with ZO-1 antibody and DAPI. **(F)** The statistical results of ZO-1 positive cells/field. **(G)** The level of LPS in the sera. **(H)** The representative immunofluorescence images of colonic sections stained with F4/80 antibody and DAPI. **(I)** The statistical results of F4/80 positive cells/field. The mRNA expression of CD68 **(J)** and proinflammatory cytokines **(K)** in the colon of mice (n = 6). **(L-O)** The levels of TNF-α and IL-6 in the serum and colon (n = 6). Scale bar 50 μm. Values are mean ± SEM.

**Table S1.** Predicted KEGG functional pathway differences at level 2 inferred from 16S rRNA gene sequences using PICRUSt.

| **KO functional categories** | | **LF mean% (SD%)** | **LFE mean% (SD%)** | **HF mean% (SD%)** | **HFE mean% (SD%)** | **LF vs HF**  **p value** | **HF vs HFE**  **p value** |
| --- | --- | --- | --- | --- | --- | --- | --- |
| **level1** | **level2** |  |  |  |  |  |  |
| Cellular Processe | Cell Growth and Death | 0.550（0.013） | 0.564(0.008) | 0.481（0.018） | 0.501（0.014） | <0.001 | - |
| Cellular Processes | Cell Motility | 1.227（0.162） | 1.331(0.255) | 3.016（0.728） | 3.319（0.933） | 0.001 | - |
| Cellular Processes | Transport and Catabolism | 0.537（0.034） | 0.526(0.039) | 0.307（0.032） | 0.299（0.025） | <0.001 | - |
| Environmental Information Processing | Membrane Transport | 9.442（0.639） | 9.231(0.542) | 13.94（0.408） | 13.046（0.23） | <0.001 | 0.002 |
| Environmental Information Processing | Signal Transduction | 1.405（0.047） | 1.417(0.074) | 1.963（0.241） | 2.039（0.276） | 0.002 | - |
| Genetic Information Processing | Folding, Sorting and Degradation | 2.690（0.051） | 2.728(0.041) | 2.259（0.047） | 2.370（0.022） | <0.001 | 0.001 |
| Genetic Information Processing | Replication and Repair | 9.463（0.119） | 9.589(0.116) | 8.262（0.395） | 8.333（0.242） | <0.001 | - |
| Genetic Information Processing | Translation | 6.113（0.120） | 6.283(0.061) | 5.236（0.301） | 5.441（0.097） | <0.001 | - |
| Genetic Information Processing | Transcription | 2.270（0.081） | 2.210(0.074) | 2.906（0.123） | 2.763（0.110） | <0.001 | - |
| Human Diseases | Infectious Diseases | 0.379（0.013） | 0.372(0.004) | 0.349（0.019） | 0.359（0.013） | 0.011 | - |
| Human Diseases | Immune System Diseases | 0.051（0.001） | 0.051(0.002) | 0.042（0.004） | 0.040（0.005） | 0.002 | - |
| Human Diseases | Metabolic Diseases | 0.134（0.004） | 0.135(0.004) | 0.092（0.008） | 0.092（0.005） | <0.001 | - |
| Metabolism | Amino Acid Metabolism | 10.339（0.142） | 10.253(0.189) | 9.214（0.214） | 9.221（0.273） | <0.001 | - |
| Metabolism | Biosynthesis of Other Secondary Metabolites | 1.096（0.032） | 1.073(0.044) | 0.885（0.039） | 0.856（0.065） | <0.001 | - |
| Metabolism | Enzyme Families | 2.289（0.029） | 2.280(0.037) | 2.187（0.083） | 2.119（0.082） | 0.029 | - |
| Metabolism | Energy Metabolism | 6.430（0.116） | 6.539(0.092) | 5.509（0.131） | 5.724（0.088） | <0.001 | 0.009 |
| Metabolism | Glycan Biosynthesis and Metabolism | 3.254（0.192） | 3.300(0.162) | 2.183（0.132） | 2.360（0.042） | <0.001 | 0.020 |
| Metabolism | Metabolism of Cofactors and Vitamins | 4.459（0.082） | 4.495(0.090) | 3.942（0.122） | 4.071（0.111） | <0.001 | - |
| Metabolism | Metabolism of Other Amino Acids | 1.650（0.023） | 1.634(0.034) | 1.439（0.049） | 1.420（0.074） | <0.001 | - |
| Metabolism | Metabolism of Terpenoids and Polyketides | 1.771（0.032） | 1.764(0.026) | 1.497（0.108） | 1.540（0.062） | 0.001 | - |
| Metabolism | Nucleotide Metabolism | 4.359（0.053） | 4.420(0.049) | 3.790（0.155） | 3.815（0.082） | <0.001 | - |
| Metabolism | Xenobiotics Biodegradation and Metabolism | 1.594（0.110） | 1.532(0.036) | 1.799（0.139） | 1.743（0.070） | 0.019 | - |
| Organismal Systems | Circulatory System | 0.002（0.001） | 0.004（0.002） | 0.003（0.002） | 0.008（0.005） | - | 0.043 |
| Organismal Systems | Digestive System | 0.039（0.004） | 0.039(0.004) | 0.017（0.003） | 0.018（0.005） | <0.001 | - |
| Organismal Systems | Environmental Adaptation | 0.135（0.003） | 0.138(0.006) | 0.172（0.010） | 0.179（0.014） | <0.001 | - |
| Organismal Systems | Endocrine System | 0.307（0.014） | 0.308(0.013) | 0.252（0.015） | 0.256（0.011） | <0.001 | - |
| Organismal Systems | Excretory System | 0.041（0.002） | 0.040(0.002) | 0.024（0.007） | 0.027（0.012） | 0.001 | - |
| Organismal Systems | Immune System | 0.100（0.004） | 0.099(0.002) | 0.082（0.005） | 0.083（0.002） | <0.001 | - |
| Unclassified | Genetic Information Processing | 2.526（0.051） | 2.585(0.037) | 2.655（0.069） | 2.768（0.076） | 0.005 | 0.022 |
| Unclassified | Metabolism | 2.568（0.013） | 2.533(0.010) | 2.700（0.056） | 2.670（0.052） | 0.002 | - |
| Unclassified | Poorly Characterized | 4.899（0.058） | 4.856(0.037) | 5.000（0.087） | 5.009（0.065） | 0.042 | - |

Note: Data is expressed as mean (%) (SD%). KEGG Kyoto Encyclopedia of Genes and Genomes, PICRUSt Phylogenetic Investigation of Communities by Reconstruction of Unobserved States, KO KEGG ortholog; SD Standard Deviation.
